# A Tunable Multivariate Metal–Organic Framework as a Platform for Designing Photocatalysts

**DOI:** 10.1021/jacs.1c01764

**Published:** 2021-04-26

**Authors:** Yang Wang, Hao Lv, Erik Svensson Grape, Carlo Alberto Gaggioli, Akhil Tayal, Aditya Dharanipragada, Tom Willhammar, A. Ken Inge, Xiaodong Zou, Ben Liu, Zhehao Huang

**Affiliations:** † College of Chemistry, Sichuan University, Chengdu 610064, China; ‡ Department of Materials and Environmental Chemistry, Stockholm University, Stockholm SE-106 91, Sweden; # Department of Chemistry, University of Chicago, Chicago, Illinois 60637, United States; ⊥ Photon Science, Deutsches Elektronen-Synchrotron, Hamburg 22607, Germany; § Key Laboratory for Soft Chemistry and Functional Materials, Nanjing University of Science and Technology, Ministry of Education, Nanjing 210094, China; ¶ Jiangsu Key Laboratory of New Power Batteries, Jiangsu Collaborative Innovation Center of Biomedical Functional Materials, School of Chemistry and Materials Science, Nanjing Normal University, Nanjing 210023, China

## Abstract

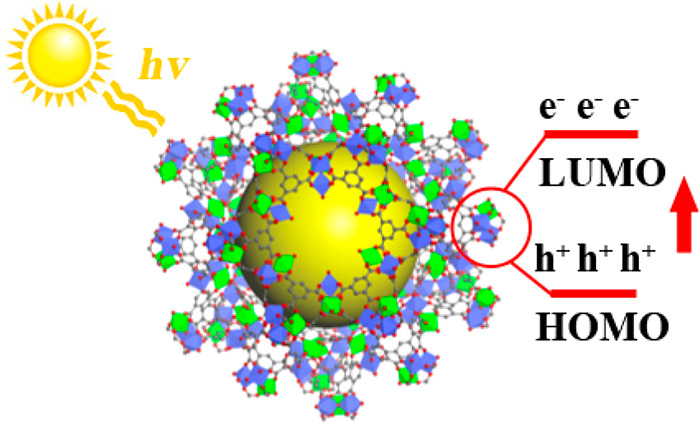

Catalysts for photochemical reactions underlie many foundations in our lives, from natural light harvesting to modern energy storage and conversion, including processes such as water photolysis by TiO_2_. Recently, metal–organic frameworks (MOFs) have attracted large interest within the chemical research community, as their structural variety and tunability yield advantages in designing photocatalysts to address energy and environmental challenges. Here, we report a series of novel multivariate metal–organic frameworks (MTV-MOFs), denoted as MTV-MIL-100. They are constructed by linking aromatic carboxylates and AB_2_OX_3_ bimetallic clusters, which have ordered atomic arrangements. Synthesized through a solvent-assisted approach, these ordered and multivariate metal clusters offer an opportunity to enhance and fine-tune the electronic structures of the crystalline materials. Moreover, mass transport is improved by taking advantage of the high porosity of the MOF structure. Combining these key advantages, MTV-MIL-100(Ti,Co) exhibits a high photoactivity with a turnover frequency of 113.7 mol_H2_ g_cat._^–1^ min^–1^, a quantum efficiency of 4.25%, and a space time yield of 4.96 × 10^–5^ in the photocatalytic hydrolysis of ammonia borane. Bridging the fields of perovskites and MOFs, this work provides a novel platform for the design of highly active photocatalysts.

Energy and the environment are among the most important issues of society nowadays. Solar energy, a clean and renewable source, can be efficiently harnessed by photocatalytic processes to tackle such challenges. The efficiency of photocatalysis in reactions such as hydrogen production and the degradation of organic molecules is highly dependent on the catalyst, which can create electron–hole pairs and generate free radicals for secondary reactions. Since the discovery of water photolysis by TiO_2_,^1^ it has become the predominant material for photocatalysis due to its high efficiency, high stability, and abundance.

Among the various materials developed as photocatalysts,^2−6^ metal–organic frameworks (MOFs) are a family of inorganic–organic hybrid materials with permanent porosity and can have extraordinarily high surface areas.^7,8^ More importantly, compared to TiO_2_-based materials, MOFs provide great opportunities in the design of building units using supramolecular and reticular chemistry.^9^ By a rational selection of metal nodes and organic linkers, the optical response, electronic structures, and binding energetics of MOFs can be tailored. These aspects make MOFs a promising class of photocatalysts for applications such as artificial photosynthesis,^10−14^ degradation of organics,^15−17^ and water oxidation.^18−21^ Moreover, multiple metals can be incorporated in a MOF structure. The resulting materials then belong to the subclass of multivariate (MTV)-MOFs, which are characterized by the possibility of having synergetic effects between different components in order to achieve optimal performance.^22−29^

In the context of photocatalytic properties, Ti-oxo clusters are attractive secondary building units. By acting as analogues of TiO_2_ nanoparticles, high chemical stability, redox activity, and photocatalytic properties are expected.^30−33^ However, the formation of Ti-oxo clusters is highly sensitive to reaction conditions, which are often incompatible with those for MOF crystallization. Consequently, less than 30 Ti-based MOFs have been reported among the more than 80 000 different MOF structures,^34^ and Ti-based MTV-MOFs are even more scarce.^29^ Doping with a second metal element is a widely adopted approach to prepare mixed-metal MOFs.^35^ However, doping is hard to control and the resulting materials often exhibit compositional gradients, e.g., from the surface to the center.

Here, we present a solvent-assisted approach that leads to a highly tunable platform for the design and synthesis of MOFs. The core of the platform is based on trinuclear clusters with a well-defined atomic structure of AB_2_OX_3_, where the transition metal ions on the A site connect with two other ions on the B sites through a bridging μ_3_-O, while X sites (−OH or H_2_O) coordinate to the A and B sites as terminal species (Figure 1). Similar to perovskites, it therefore offers great advantages over conventional materials such as metal oxides and carbon-based materials for fine-tuning the acquired compositions and corresponding electronic structures for a wide range of catalysis. To focus on the photocatalytic applications, we present a series of trinuclear clusters featuring Ti(IV) in the A site. Different transition metals (Co, Ni, Mn, Fe) residing in the B sites are used for fine-tuning the electronic structure and enhancing catalytic cooperativity.

**Figure 1:**
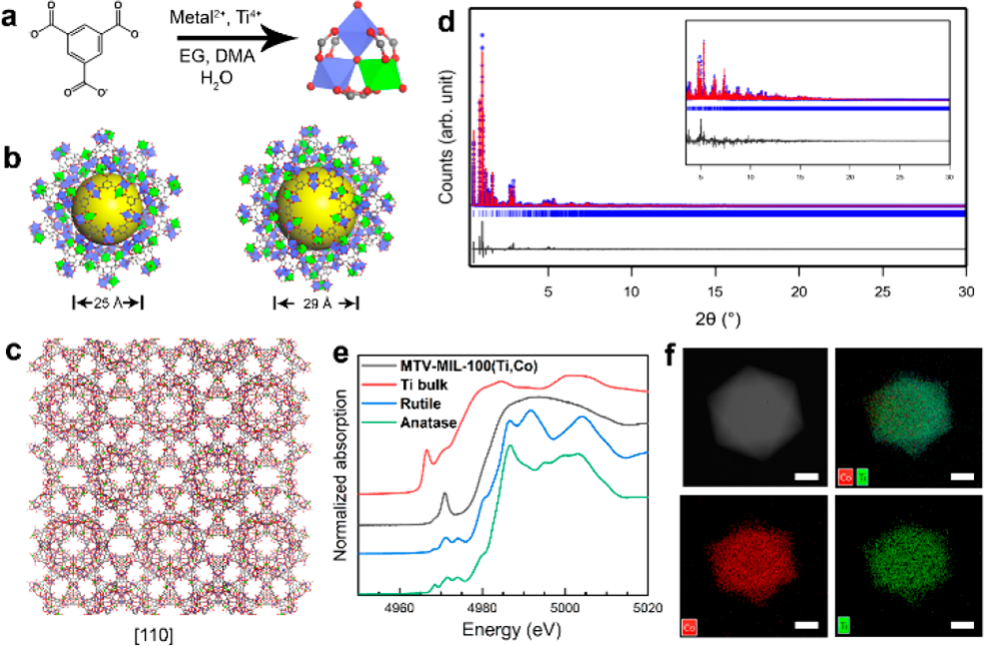
(a) Synthetic scheme of AB_2_OX_3_ clusters. (b) Structural representation of two types of mesoporous cages in MTV-MIL-100(Ti,Co). (c) Structural model of MTV-MIL-100(Ti,Co) viewed along the [110] direction. Green octahedra: Ti; blue octahedra: Co; red spheres: O; gray spheres: C. (d) Rietveld refinement of MTV-MIL-100(Ti,Co) against synchrotron PXRD data (λ = 0.412 836 Å). (e) XANES spectra of MTV-MIL-100(Ti,Co) compared with Ti references. (f) EDS mapping of MTV-MIL-100(Ti,Co), showing a homogeneous Ti and Co distribution (scale bar = 1 μm).

Specifically, the reaction was carried out in a solvent mixture of ethylene glycol (EG) and *N*,*N*-dimethylacetamide (DMA). As EG can simultaneously coordinate to A and B cations, it offers protection against moisture and prevents A and B cations from undergoing an otherwise rapid hydrolysis, which would result in dense phases. The high excess of EG (EG/B^2+^ = 1800, molar ratio) also serves as a competing reagent to 1,3,5-benzenetricarboxylate (BTC) linkers to control reaction kinetics and crystal growth. In addition, due to the high EG content, which leads to an acidic environment, X sites are favored to be H_2_O instead of OH^–^. This promotes the ordered organization of A and B cations to minimize the charge during the formation of AB_2_OX_3_ clusters (Figures S1–S5). A low amount of EG resulted in smaller crystal sizes and lower crystallinities (Figure S6). After linking the clusters with BTCs, the resulting materials exhibit an octahedral morphology with a 2–5 μm diameter (Figures S7, S8 and Movie S1). The powder X-ray diffraction (PXRD) pattern shows sharp and intense peaks indicating a high crystallinity (Figures 1 and S9). The MOF crystallized in a large cubic unit cell with *a* = 73.6449(1) Å. Rietveld refinement against synchrotron PXRD data (Figure 1d and Table S1) revealed that the crystals have a MIL-100 topology^36^ (denoted as MTV-MIL-100(Ti,B)), with pore diameters of 25–30 Å (Figure 1b and c).

Elemental analysis shows a constant A:B molar ratio of 1:2 in a wide range of starting ratios (Figures 1f and S2–S5), which confirms the consistent formation of AB_2_OX_3_ clusters. X-ray absorption near edge structure (XANES) and X-ray absorption spectroscopy (XAS) analyses were performed to further understand the local coordination environment of the AB_2_OX_3_ clusters. As shown in Figures 1e and S10, MTV-MIL-100(Ti,Co) has a distinct XANES spectrum, and the pre-edge peaks, when compared to bulk Ti-metal, rutile, and anatase references, indicate a different coordination environment. Specifically, the asymmetric peak in the low-energy region (Figure S10, indicated by A1) and the absence of the high-energy peak (Figure S10, indicated by B) suggest a distorted coordination geometry,^37,38^ which resulted from the removal of terminally bonded solvent X in the AB_2_OX_3_ clusters. Density-functional theory (DFT) calculations were further applied to analyze the coordination geometry of Ti(IV). The results show that the Ti(IV) ion is closer to the central oxygen atom, causing distortions from an octahedral geometry (Figure S11). The dihedral angle between Ti(IV) and three of the four O atoms of the formate groups is ∼15°, which is deviated from 0°, as would be expected in an ideal octahedral coordination geometry. Moreover, the continuous Cauchy wavelet transform (CCWT) of the spectra shows an asymmetric wavelet for the MTV-MIL-100(Ti,Co) local environment, which confirms the heterogeneity of the cluster in the Ti–O–Co linkage (Figures S12–S14). In addition to the XAS analysis, Raman spectroscopy also indicated that the metal–metal linkages differ significantly from those in single-component crystals, such as MIL-125(Ti) (Figure S15).

The strong interaction between Ti(IV) and O atoms endows MTV-MIL-100(Ti,Co) with high chemical stability as its structure remains intact after being treated with highly acidic and basic aqueous solutions (Figure 2a). The permanent porosity of MTV-MIL-100(Ti,B) was confirmed by the analysis of N_2_ sorption isotherms (Figures S16–S19). MTV-MIL-100(Ti,Co) shows a high Brunauer–Emmett–Teller (BET) surface area of 1695 m^2^ g^–1^ (Figure 2b). Notably, each AB_2_OX_3_ cluster in MTV-MIL-100 is accessible via their mesopores (Figure 1b), which offers a much better mass-transport pathway compared to traditional photocatalysts such as TiO_2_. Furthermore, the terminal X sites can cleave from the cluster upon activation, leaving open metal sites for binding reaction species. The ordered AB_2_OX_3_ cluster offers a platform to adjust the electronic structure of the resulting MOF. As calculated from ultraviolet–visible (UV–vis) spectra (Figure 2c), MTV-MIL-100(Ti,Co) has an optical band gap of 2.7 eV, which is within the range for visible light. By further altering the transition metal ions of the B sites, the optical band gap can be tuned from 2.3 eV to 3.1 eV (Figure S20). The composition of MTV-MIL-100(Ti,Co) was also confirmed by X-ray photoelectron spectroscopy (XPS) analysis, which further indicated the presence of both Co(II) and Co(III) sites, together with Ti(IV) in the cluster (Figure 2d). This agrees well with the electron energy-loss spectroscopy (EELS) analysis (Figure S21). In addition, the structure of MTV-MIL-100(Ti,Co) is stable up to 360 °C (Figures S22 and S23).

**Figure 2:**
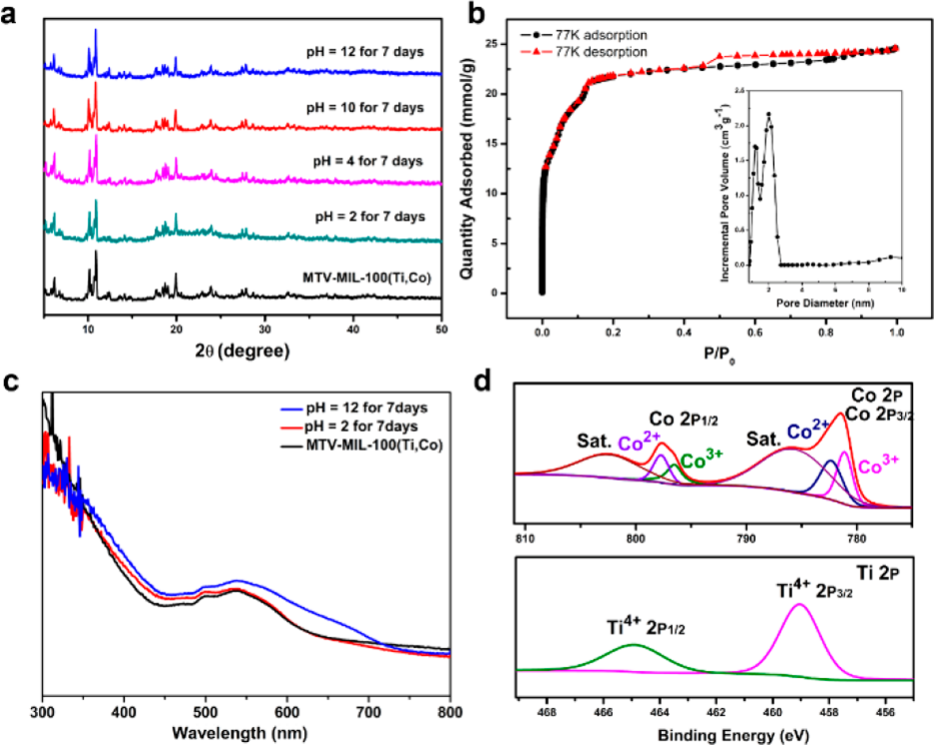
(a) PXRD patterns of MTV-MIL-100(Ti,Co) treated in different pH solutions (λ = 1.5416 Å). (b) N_2_ sorption isotherm of MTV-MIL-100(Ti,Co). (c) UV–vis spectra of MTV-MIL-100(Ti,Co) after treatment in different pH solutions, showing a band gap of ∼2.7 eV. (d) High-resolution XPS Co 2p and Ti 2p spectra of MTV-MIL-100(Ti,Co).

To gain insight into the photocatalytic activity of the MTV-MIL-100(Ti,Co), its electronic structure was investigated by computational methods using truncated models (Figures S11 and S24). The calculation of molecular orbital diagrams was based on an octet spin state, which was found as the most stable spin state (Table S2). The HOMO is mostly a d orbital with β spin localized on Co(II), and the energy of the HOMO was set to be the zero reference. As shown in Figure 3, the molecular orbitals diagram therefore indicates a metal-to-metal charge transfer (MMCT) from a Co(II) to a Co(III) (from the HOMO to the LUMO), producing separation of charges that can give rise to the photocatalytic activity.^39−42^ The transition is located at 2.54 eV, which is in the visible light range and in good agreement with the experimental value of 2.7 eV. The MMCT from Co(II) to Ti(IV) is located at 3.51–3.68 eV. Considering the uncertainty of the method, this transition could also lie in the visible range. The ligand-to-metal charge transfer (LMCT) is located at higher energies, 3.57 eV for BTC to Co(III) and 4.54–4.71 eV for BTC to Ti(IV), which should be an unfavorable transition due to their higher spatial separation. Moreover, TDDFT calculation shows that the BTC local transition is located at 5.77 eV (Figure S25), which is much higher than the visible light range. Illuminating MTV-MIL-100(Ti,Co) results in an intense paramagnetic signal in the paramagnetic resonance (EPR) spectra (Figure S26), which is attributed to the MMCT between Co–Co and Co–Ti.

**Figure 3:**
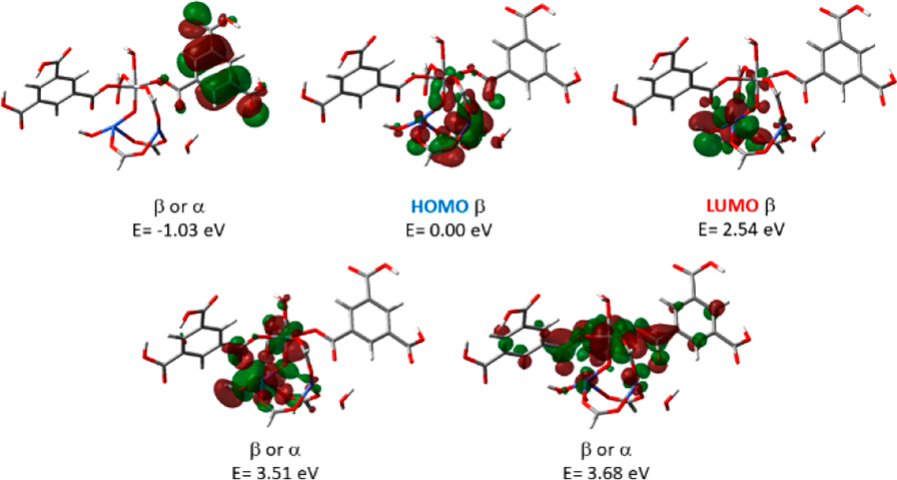
Molecular orbital diagrams for MTV-MIL-100(Ti,Co).

With the ultrahigh surface area, which provides high accessibility within the crystals, tunable electronic structures, which offers an MMCT route to control the optical response, and excellent water resistance, which prevents catalyst degradation, MTV-MIL-100(Ti,B) presents an ideal example for designing and building photocatalysts. As a proof of concept, the photochemical hydrolysis of ammonia borane (AB) was tested (Figures S27 and S28). Figure 4a shows the time-dependent plots of H_2_ production catalyzed by MTV-MIL-100(Ti,Co), MIL-125(Ti), and commercial TiO_2_ (P25) in the presence of visible light (360–780 nm). MTV-MIL-100(Ti,Co) displays the highest reaction kinetics. It takes only 6 min to complete H_2_ production, with a high turnover frequency (TOF) of 113.7 mol_H2_ g_cat._^–1^ min^–1^, compared to the TOF values of MIL-125(Ti) and P25 of 2.4 and 8.6 mol_H2_ g_cat._^–1^ min^–1^, respectively. MTV-MIL-100(Ti,Co) also exhibits a higher quantum efficiency (QE) of 4.25%, space time yield (STY) of 4.96 × 10^–5^, and figure of merit (FOM) of 2.43 × 10^–6^ than those of MIL-125(Ti) and P25 (Table 1; see the Supporting Information for more details). Moreover, MTV-MIL-100(Ti,Co) represents one of the best photocatalysts in comparison with other state-of-the-art catalysts (Table S3).

**Table 1:** Comparisons of Photocatalytic Activity of MTV-MIL-100(Ti,Co), MIL-125, and P25

catalyst^a^	band gap (eV)^b^	reaction efficiency (%)^c^	TOF (mol_H2_ g_cat._^–1^ min^–1^)^d^	QE (%)^d^^,^^e^	STY (10^–5^)^d^^,^^e^	FOM (10^–7^)^d^^,^^e^
MTV-MIL-100 (Ti,Co)	2.7	100	113.7	4.25	4.96	24.3
MIL-125(Ti)	3.2	1.4	2.4	2.94	1.85	4.07
P25(TiO_2_)	2.9	5.2	8.6	1.78	3.41	14.8

a With 1.5 wt % of Pt as cocatalyst.

b Calculated based on UV–vis spectra.

c Based on the reaction time of 10 min.

d Reaction conditions: AB concentration = 2.86 mg mL^–1^; light power = 300 J s^–1^; irradiation time = 20 min; catalyst dose = 2.4 mg (0.6 mg mL^–1^).

e See Supporting Information for calculation details.

**Figure 4:**
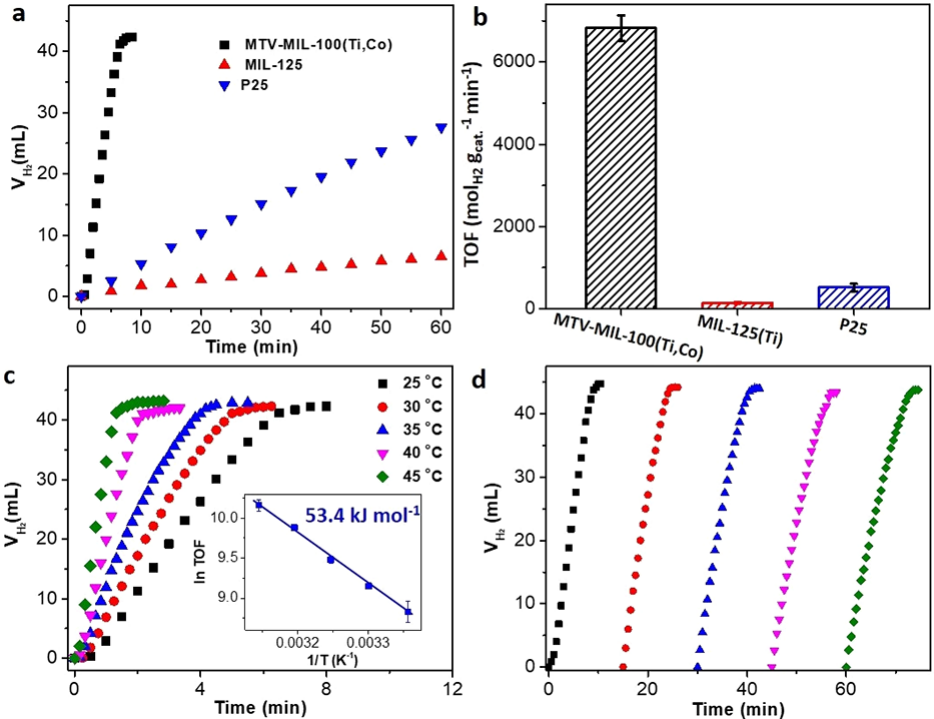
(a) Catalytic activities and (b) calculated TOF values in the presence of visible light. (c) Catalytic activities and corresponding Arrhenius plots of MTV-MIL-100(Ti,Co) collected in different testing temperatures. (d) Catalytic stability of MTV-MIL-100(Ti,Co) for five runs. 1.5 wt % of Pt was used as a cocatalyst.

We further studied the catalytic activities of MTV-MIL-100(Ti,Co) in different temperatures to investigate reaction kinetics (Figure 4c). MTV-MIL-100(Ti,Co) shows an enhanced photocatalytic activity with an increase of reaction temperature. Normalizing the relationships between ln(TOF) and 1/*T*, the linear slope confirms that the hydrolysis of AB over MTV-MIL-100(Ti,Co) follows the Arrhenius equation (Figure 4c). The activation energy (*E*_a_) was calculated as 53.4 kJ mol^–1^, indicating a low-energy barrier to promote the hydrolysis of AB. Meanwhile, MTV-MIL-100(Ti,Co) also displays good catalytic stability (Figure 4d). After five runs, only 10.5 min was needed for the complete hydrolysis of AB with a TOF of 64.8 mol_H2_ g_cat._^–1^ min^–1^. The decreased TOF is mainly due to catalyst deactivation and dilution of the reactants.^43,44^ In addition, using a higher Pt loading could further improve the performance, and the TOF reaches a maximum of 136.4 mol_H2_ g_cat._^–1^ min^–1^ by loading at least 2.0 wt % of Pt (Figure S29). Postcatalytic characterization indicates that the crystal structure of MTV-MIL-100(Ti,Co) remains intact, without any obvious degradation or phase transitions occurring (Figure S30).

In conclusion, we have developed a series of photocatalysts based on the AB_2_OX_3_ cluster, which have well-defined heterostructures that provide tunability of the electronic structures and synergetic cooperation for photocatalysis through MMCT. The resulting materials are among the rare examples of MTV-MOFs with ordered spatial arrangements of the multiple metal ions.^45^ In addition to the structural tunability, MTV-MIL-100(Ti,Co) exhibit high porosity and water stability. Due to these advantages, it demonstrates high photocatalytic activity compared to the pure Ti-MOF MIL-125 and commercial TiO_2_. Our solvent-assisted synthetic strategy when making MTV-MIL-100 presents a facile way of controlling the composition of the material. Considering the large compositional diversity, as well as the huge library of organic linkers available, we believe that there are many discoveries to be made by utilizing AB_2_OX_3_ clusters for constructing inorganic–organic hybrid materials, especially in the development of even more efficient photocatalysts.

## Supporting Information Available

Movie S1: 3D tomography of MTV-MIL-100(Ti,Co) (MP4)
Experimental details, PXRD, gas sorption, SEM, structure refinement, XPS, photocatalytic H_2_ production, and DFT calculations (PDF)

## Accession Codes

CCDC 2031951 contains the supplementary crystallographic data for this paper. These data can be obtained free of charge via www.ccdc.cam.ac.uk/data_request/cif, or by emailing data_request@ccdc.cam.ac.uk, or by contacting The Cambridge Crystallographic Data Centre, 12 Union Road, Cambridge CB2 1EZ, UK; fax: +44 1223 336033.

## References

[ref1] Fujishima A.; Honda K. Electrochemical Photolysis of Water at a Semiconductor Electrode. Nature 1972, 238 (5358), 37–38. 10.1038/238037a0. 12635268

[ref2] Hwang J.; Rao R. R.; Giordano L.; Katayama Y.; Yu Y.; Shao-Horn Y. Perovskites in Catalysis and Electrocatalysis. Science 2017, 358 (6364), 751–756. 10.1126/science.aam7092. 29123062

[ref3] Wang W.; Tadé M. O.; Shao Z. Research Progress of Perovskite Materials in Photocatalysis- and Photovoltaics-Related Energy Conversion and Environmental Treatment. Chem. Soc. Rev. 2015, 44 (15), 5371–5408. 10.1039/C5CS00113G. 25976276

[ref4] Huang P.; Huang J.; Pantovich S. A.; Carl A. D.; Fenton T. G.; Caputo C. A.; Grimm R. L.; Frenkel A. I.; Li G. Selective CO2 Reduction Catalyzed by Single Cobalt Sites on Carbon Nitride under Visible-Light Irradiation. J. Am. Chem. Soc. 2018, 140 (47), 16042–16047. 10.1021/jacs.8b10380. 30415539

[ref5] Anwer H.; Mahmood A.; Lee J.; Kim K.-H.; Park J.-W.; Yip A. C. K. Photocatalysts for Degradation of Dyes in Industrial Effluents: Opportunities and Challenges. Nano Res. 2019, 12 (5), 955–972. 10.1007/s12274-019-2287-0.

[ref6] Zhang W.; Tian Y.; He H.; Xu L.; Li W.; Zhao D. Recent Advances in the Synthesis of Hierarchically Mesoporous TiO2Materials for Energy and Environmental Applications. Natl. Sci. Rev. 2020, 7 (11), 1702–1725. 10.1093/nsr/nwaa021. PMC8288798 34691503

[ref7] Yaghi O. M.; O’Keeffe M.; Ockwig N. W.; Chae H. K.; Eddaoudi M.; Kim J. Reticular Synthesis and the Design of New Materials. Nature 2003, 423 (6941), 705–714. 10.1038/nature01650. 12802325

[ref8] Kitagawa S.; Kitaura R.; Noro S. Functional Porous Coordination Polymers. Angew. Chem., Int. Ed. 2004, 43 (18), 2334–2375. 10.1002/anie.200300610. 15114565

[ref9] Eddaoudi M.; Kim J.; Rosi N.; Vodak D.; Wachter J.; O’Keeffe M.; Yaghi O. M. Systematic Design of Pore Size and Functionality in Isoreticular MOFs and Their Application in Methane Storage. Science 2002, 295 (5554), 469–472. 10.1126/science.1067208. 11799235

[ref10] Wu P.; He C.; Wang J.; Peng X.; Li X.; An Y.; Duan C. Photoactive Chiral Metal–Organic Frameworks for Light-Driven Asymmetric α-Alkylation of Aldehydes. J. Am. Chem. Soc. 2012, 134 (36), 14991–14999. 10.1021/ja305367j. 22888952

[ref11] Elcheikh Mahmoud M.; Audi H.; Assoud A.; Ghaddar T. H.; Hmadeh M. Metal–Organic Framework Photocatalyst Incorporating Bis(4′-(4-Carboxyphenyl)-Terpyridine)Ruthenium(II) for Visible-Light-Driven Carbon Dioxide Reduction. J. Am. Chem. Soc. 2019, 141 (17), 7115–7121. 10.1021/jacs.9b01920. 30974057

[ref12] Li N.; Liu J.; Liu J.-J.; Dong L.-Z.; Xin Z.-F.; Teng Y.-L.; Lan Y.-Q. Adenine Components in Biomimetic Metal–Organic Frameworks for Efficient CO2 Photoconversion. Angew. Chem., Int. Ed. 2019, 58 (16), 5226–5231. 10.1002/anie.201814729. 30656814

[ref13] Qiao G.-Y.; Yuan S.; Pang J.; Rao H.; Lollar C. T.; Dang D.; Qin J.-S.; Zhou H.-C.; Yu J. Functionalization of Zirconium-Based Metal–Organic Layers with Tailored Pore Environments for Heterogeneous Catalysis. Angew. Chem., Int. Ed. 2020, 59 (41), 18224–18228. 10.1002/anie.202007781. 32613736

[ref14] Pang J.; Di Z.; Qin J.-S.; Yuan S.; Lollar C. T.; Li J.; Zhang P.; Wu M.; Yuan D.; Hong M.; Zhou H.-C. Precisely Embedding Active Sites into a Mesoporous Zr-Framework through Linker Installation for High-Efficiency Photocatalysis. J. Am. Chem. Soc. 2020, 142 (35), 15020–15026. 10.1021/jacs.0c05758. 32786762

[ref15] Laurier K. G. M.; Vermoortele F.; Ameloot R.; De Vos D. E.; Hofkens J.; Roeffaers M. B. J. Iron(III)-Based Metal–Organic Frameworks As Visible Light Photocatalysts. J. Am. Chem. Soc. 2013, 135 (39), 14488–14491. 10.1021/ja405086e. 24015906

[ref16] Wang C.-C.; Li J.-R.; Lv X.-L.; Zhang Y.-Q.; Guo G. Photocatalytic Organic Pollutants Degradation in Metal–Organic Frameworks. Energy Environ. Sci. 2014, 7 (9), 2831–2867. 10.1039/C4EE01299B.

[ref17] Gao Q.; Xu J.; Bu X.-H. Recent Advances about Metal–Organic Frameworks in the Removal of Pollutants from Wastewater. Coord. Chem. Rev. 2019, 378, 17–31. 10.1016/j.ccr.2018.03.015.

[ref18] Wang C.; Xie Z.; deKrafft K. E.; Lin W. Doping Metal–Organic Frameworks for Water Oxidation, Carbon Dioxide Reduction, and Organic Photocatalysis. J. Am. Chem. Soc. 2011, 133 (34), 13445–13454. 10.1021/ja203564w. 21780787

[ref19] Nepal B.; Das S. Sustained Water Oxidation by a Catalyst Cage-Isolated in a Metal–Organic Framework. Angew. Chem., Int. Ed. 2013, 52 (28), 7224–7227. 10.1002/anie.201301327. 23729244

[ref20] Gong Y.-N.; Ouyang T.; He C.-T.; Lu T.-B. Photoinduced Water Oxidation by an Organic Ligand Incorporated into the Framework of a Stable Metal–Organic Framework. Chem. Sci. 2016, 7 (2), 1070–1075. 10.1039/C5SC02679B. 28936323 PMC5590092

[ref21] Liang Z.; Qu C.; Guo W.; Zou R.; Xu Q. Pristine Metal–Organic Frameworks and Their Composites for Energy Storage and Conversion. Adv. Mater. 2018, 30 (37), 1702891 10.1002/adma.201702891. 29164712

[ref22] Zheng S.-T.; Wu T.; Zuo F.; Chou C.; Feng P.; Bu X. Mimicking Zeolite to Its Core: Porous Sodalite Cages as Hangers for Pendant Trimeric M3(OH) Clusters (M = Mg, Mn, Co, Ni, Cd). J. Am. Chem. Soc. 2012, 134 (4), 1934–1937. 10.1021/ja209800x. 22280215

[ref23] Wang L. J.; Deng H.; Furukawa H.; Gándara F.; Cordova K. E.; Peri D.; Yaghi O. M. Synthesis and Characterization of Metal–Organic Framework-74 Containing 2, 4, 6, 8, and 10 Different Metals. Inorg. Chem. 2014, 53 (12), 5881–5883. 10.1021/ic500434a. 24878113

[ref24] Cui Y.; Xu H.; Yue Y.; Guo Z.; Yu J.; Chen Z.; Gao J.; Yang Y.; Qian G.; Chen B. A Luminescent Mixed-Lanthanide Metal–Organic Framework Thermometer. J. Am. Chem. Soc. 2012, 134 (9), 3979–3982. 10.1021/ja2108036. 22352469

[ref25] Tu B.; Pang Q.; Wu D.; Song Y.; Weng L.; Li Q. Ordered Vacancies and Their Chemistry in Metal–Organic Frameworks. J. Am. Chem. Soc. 2014, 136 (41), 14465–14471. 10.1021/ja5063423. 25229624

[ref26] Liu Q.; Cong H.; Deng H. Deciphering the Spatial Arrangement of Metals and Correlation to Reactivity in Multivariate Metal–Organic Frameworks. J. Am. Chem. Soc. 2016, 138 (42), 13822–13825. 10.1021/jacs.6b08724. 27701854

[ref27] Fracaroli A. M.; Siman P.; Nagib D. A.; Suzuki M.; Furukawa H.; Toste F. D.; Yaghi O. M. Seven Post-Synthetic Covalent Reactions in Tandem Leading to Enzyme-like Complexity within Metal–Organic Framework Crystals. J. Am. Chem. Soc. 2016, 138 (27), 8352–8355. 10.1021/jacs.6b04204. 27346625 PMC5376101

[ref28] Xia Q.; Li Z.; Tan C.; Liu Y.; Gong W.; Cui Y. Multivariate Metal–Organic Frameworks as Multifunctional Heterogeneous Asymmetric Catalysts for Sequential Reactions. J. Am. Chem. Soc. 2017, 139 (24), 8259–8266. 10.1021/jacs.7b03113. 28537723

[ref29] Castells-Gil J.; Padial N. M.; Almora-Barrios N.; Gil-San-Millán R.; Romero-Ángel M.; Torres V.; da Silva I.; Vieira B. C. J.; Waerenborgh J. C.; Jagiello J.; Navarro J. A. R.; Tatay S.; Martí-Gastaldo C. Heterometallic Titanium-Organic Frameworks as Dual-Metal Catalysts for Synergistic Non-Buffered Hydrolysis of Nerve Agent Simulants. Chem. 2020, 6, 3118–3131. 10.1016/j.chempr.2020.09.002.

[ref30] Dan-Hardi M.; Serre C.; Frot T.; Rozes L.; Maurin G.; Sanchez C.; Férey G. A New Photoactive Crystalline Highly Porous Titanium(IV) Dicarboxylate. J. Am. Chem. Soc. 2009, 131 (31), 10857–10859. 10.1021/ja903726m. 19621926

[ref31] Nguyen H. L.; Gándara F.; Furukawa H.; Doan T. L. H.; Cordova K. E.; Yaghi O. M. A Titanium–Organic Framework as an Exemplar of Combining the Chemistry of Metal– and Covalent–Organic Frameworks. J. Am. Chem. Soc. 2016, 138 (13), 4330–4333. 10.1021/jacs.6b01233. 26998612

[ref32] Yuan S.; Qin J.-S.; Xu H.-Q.; Su J.; Rossi D.; Chen Y.; Zhang L.; Lollar C.; Wang Q.; Jiang H.-L.; Son D. H.; Xu H.; Huang Z.; Zou X.; Zhou H.-C. [Ti8Zr2O12(COO)16] Cluster: An Ideal Inorganic Building Unit for Photoactive Metal–Organic Frameworks. *ACS Cent*. ACS Cent. Sci. 2018, 4 (1), 105–111. 10.1021/acscentsci.7b00497. 29392182 PMC5785768

[ref33] Castells-Gil J.; Padial N. M.; Almora-Barrios N.; da Silva I.; Mateo D.; Albero J.; García H.; Martí-Gastaldo C. De Novo Synthesis of Mesoporous Photoactive Titanium(IV)–Organic Frameworks with MIL-100 Topology. Chem. Sci. 2019, 10 (15), 4313–4321. 10.1039/C8SC05218B. 31057758 PMC6472189

[ref34] Groom C. R.; Bruno I. J.; Lightfoot M. P.; Ward S. C. The Cambridge Structural Database. Acta Crystallogr., Sect. B: Struct. Sci., Cryst. Eng. Mater. 2016, 72 (2), 171–179. 10.1107/S2052520616003954. PMC4822653 27048719

[ref35] Abednatanzi S.; Derakhshandeh P. G.; Depauw H.; Coudert F.-X.; Vrielinck H.; Voort P. V. D.; Leus K. Mixed-Metal Metal–Organic Frameworks. Chem. Soc. Rev. 2019, 48 (9), 2535–2565. 10.1039/C8CS00337H. 30989162

[ref36] Férey G.; Serre C.; Mellot-Draznieks C.; Millange F.; Surblé S.; Dutour J.; Margiolaki I. A Hybrid Solid with Giant Pores Prepared by a Combination of Targeted Chemistry, Simulation, and Powder Diffraction. Angew. Chem., Int. Ed. 2004, 43 (46), 6296–6301. 10.1002/anie.200460592. 15372634

[ref37] Babonneau F.; Doeuff S.; Leaustic A.; Sanchez C.; Cartier C.; Verdaguer M. XANES and EXAFS Study of Titanium Alkoxides. Inorg. Chem. 1988, 27 (18), 3166–3172. 10.1021/ic00291a024.

[ref38] Jiang N.; Su D.; Spence J. C. H. Determination of Ti Coordination from Pre-Edge Peaks in Ti K -Edge XANES. Phys. Rev. B: Condens. Matter Mater. Phys. 2007, 76 (21), 1–9. 10.1103/PhysRevB.76.214117.

[ref39] Liu H.; Xu C.; Li D.; Jiang H.-L. Photocatalytic Hydrogen Production Coupled with Selective Benzylamine Oxidation over MOF Composites. Angew. Chem. 2018, 130 (19), 5477–5481. 10.1002/ange.201800320. 29508919

[ref40] Campanelli M.; Del Giacco T.; De Angelis F.; Mosconi E.; Taddei M.; Marmottini F.; D’Amato R.; Costantino F. Solvent-Free Synthetic Route for Cerium(IV) Metal–Organic Frameworks with UiO-66 Architecture and Their Photocatalytic Applications. ACS Appl. Mater. Interfaces 2019, 11 (48), 45031–45037. 10.1021/acsami.9b13730. 31702892

[ref41] Mancuso J. L.; Hendon C. H. Titanium(IV) Inclusion as a Versatile Route to Photoactivity in Metal–Organic Frameworks. Adv. Theory Simul. 2019, 2 (11), 1900126 10.1002/adts.201900126.

[ref42] Wu X.-P.; Gagliardi L.; Truhlar D. G. Cerium Metal–Organic Framework for Photocatalysis. J. Am. Chem. Soc. 2018, 140 (25), 7904–7912. 10.1021/jacs.8b03613. 29807431

[ref43] Chen W.; Ji J.; Feng X.; Duan X.; Qian G.; Li P.; Zhou X.; Chen D.; Yuan W. Mechanistic Insight into Size-Dependent Activity and Durability in Pt/CNT Catalyzed Hydrolytic Dehydrogenation of Ammonia Borane. J. Am. Chem. Soc. 2014, 136 (48), 16736–16739. 10.1021/ja509778y. 25405630

[ref44] Ren X.; Lv H.; Yang S.; Wang Y.; Li J.; Wei R.; Xu D.; Liu B. Promoting Effect of Heterostructured NiO/Ni on Pt Nanocatalysts toward Catalytic Hydrolysis of Ammonia Borane. J. Phys. Chem. Lett. 2019, 10 (23), 7374–7382. 10.1021/acs.jpclett.9b03080. 31725303

[ref45] Ji Z.; Li T.; Yaghi O. M. Sequencing of Metals in Multivariate Metal-Organic Frameworks. Science 2020, 369 (6504), 674–680. 10.1126/science.aaz4304. 32764067

