# Supplementary material for: A Tunable Multivariate Metal–Organic Framework as a Platform for Designing Photocatalysts
Source: J Am Chem Soc. 2021 Apr 26;143(17):6333–8. doi: 10.1021/jacs.1c01764 (PMC8297731; doi:10.1021/jacs.1c01764)
Supplement: Supplementary file 2 — ja1c01764_si_002.pdf [file ja1c01764_si_002.pdf]

Supporting Information for

A Tunable Multivariate Metal-Organic Framework as A Platform for Designing Photocatalysts

Yang Wang,[+,§,&] Hao Lv,[+,&] Erik Svensson Grape,[‡] Carlo Alberto Gaggioli,[#] Akhil Tayal,[⊥] Aditya Dharanipragada,[‡] Tom Willhammar,[‡] A. Ken Inge,[‡] Xiaodong Zou,[‡] Ben Liu[+,$,]* and Zhehao Huang[‡,]*

[+]College of Chemistry, Sichuan University, Chengdu 610064, China

[‡]Department of Materials and Environmental Chemistry, Stockholm University, Stockholm SE-106 91, Sweden

[#]Department of Chemistry, University of Chicago, Chicago, Illinois 60637, United States

[⊥]Photon Science, Deutsches Elektronen-Synchrotron, Hamburg 22607, Germany

[§]Key Laboratory for Soft Chemistry and Functional Materials, Nanjing University of Science and Technology, Ministry of Education, Nanjing 210094, China

[$]Jiangsu Key Laboratory of New Power Batteries, Jiangsu Collaborative Innovation Center of Biomedical Functional Materials, School of Chemistry and Materials Science, Nanjing Normal University, Nanjing 210023, China

[&]Y.W. and H.L. contributed equally to this work.

Table of Contents:

Section 1. Materials and instrumentations

Section 2. Synthesis of MTV-MIL-100(Ti, B)

Section 3. Synthetic route of AB_2OX_3 cluster and MTV-MIL-100(A,B)

Section 4. XANES and XAS analysis

Section 5. Computational study

Section 6. Photocatalytic H_2 production

Section 1. Materials and instrumentations

Materials.

All the reagents were purchased from Sinopharm Chemical Reagent Co. Ltd. (Shanghai) and utilized as received without further purification.

Methods.

Transmission electron microscopy (TEM). The TEM studies were carried out using a JEOL 2010 transmission electron microscope with an accelerating voltage of 200 kV. Images were recorded with a Gatan CCD camera (resolution 4000 x 2700 pixels, pixel size 9 x 9 μm). TEM samples were prepared by casting a suspension of the samples on a carbon coated copper grid (300 mesh).

Scanning TEM (STEM) energy-dispersive X-ray spectroscopy (EDS). STEM-EDS maps were collected on an FEI, Talos F200X apparatus at an accelerating voltage of 200 kV, which is equipped with STEM and EDS detectors for elemental mapping analysis.

Electron energy-loss spectroscopy (EELS) analysis. EELS data were collected on an aberration-corrected Thermo Fisher Themis Z transmission electron microscope equipped with a Quantum Gatan Imaging Filter. The instrument was operated in STEM mode at an acceleration voltage of 300 kV. The EELS data was obtained in dual-EELS mode and the spectrometer was set to a dispersion of 0.25 eV/channel. The low-loss spectrum was used to correct for energy shifts.

Scanning electron microscopy (SEM). The SEM images were collected with using a JEOL JSM-7600F field emission scanning electron microscope.

Powder X-ray diffraction (PXRD). PXRD patterns were recorded on powder samples using a D/max 2500 VL/PC diffractometer (Japan) equipped with graphite-monochromatized Cu Kα radiation in 2θ ranging from 5° to 50°. Related work voltage and current were 40 kV and 100 mA, respectively.

X-ray photoelectron spectroscopy (XPS). The XPS spectra were performed on a scanning X-ray microprobe (Thermo ESCALAB 250Xi) that uses Al Kα radiation. The binding energy of the C 1s peak (284.8 eV) was employed as a standard to calibrate the binding energies of other elements (Ni and Co et al.).

Thermogravimetric analysis (TGA). The TGA was measured by using a NETZSCH STA 449 F3 thermogravimetric analyzer with a heating rate of 10 ^oC min^{-1}.

Nitrogen (N_2) sorption isotherms. The N_2 sorption isotherms were collected using an ASAP 2020 Plus (Micromeritics). Before tests, the samples were immersed in ethanol for 3 days, under which the solvent was decanted and freshly replenished for at least 3 times. The MOFs were further activated at 120 ^oC under vacuum.

Raman spectroscopy. The Raman spectra were obtained on Renishaw Invia Raman Microscope.

Fourier transform infrared (FT-IR) spectroscopy. The FT-IR spectra were recorded on a Bruker Vertex 70 Fourier transform infrared spectrometer.

Ultraviolet–visible (UV-vis) spectroscopy. The UV-vis spectra were collected with a diffuse reflectance apparatus attached on Shimadzu UV-2450 spectrophotometer.

Electron paramagnetic resonance (EPR) spectroscopy. The EPR spectra were recorded using a Bruker EMX CW microspectrometer with the perpendicular mode at room temperature.

Quantum efficiency (QE) analysis. The photocatalytic experiments were performed by a hydrogen production activity evaluation system (CEL-SPH2N, CEAULight, China) in a 300 mL Pyrex flask with a silicone rubber septum.

Section 2. Synthesis of MTV-MIL-100(Ti, B)

Synthesis of MTV-MIL-100(Ti, Co). A typical synthesis was carried out by dissolving 0.40 mmol of benzene-1,3,5-tricarboxylic acid and 0.60 mmol of $Co(NO_3)_2 \cdot 6H_2O$ in a mixture of 60 mL of ethylene glycol and 10 mL of *N*,*N*-dimethylacetamide. 5.0-89.0 μL of titanium isopropoxide (TIPO) was added under ultrasonication. After forming a clear solution, it was transferred into a 100 mL Teflon-lined stainless-steel autoclave and maintained at 150 ^{o}C for 3-4 h. Finally, the product was harvested by centrifugation, washed by ethanol, followed by drying under dynamic vacuum at room temperature.

Synthesis of MTV-MIL-100(Ti, Ni). A typical synthesis was carried out by dissolving 0.40 mmol of benzene-1,3,5-tricarboxylic acid and 0.60 mmol of $Ni(NO_3)_2 \cdot 6H_2O$ in a mixture of 60 mL of ethylene glycol and 10 mL of *N*,*N*-dimethylacetamide. 89.0 μL of TIPO was added under ultrasonication. After forming a clear solution, it was transferred into a 100 mL Teflon-lined stainless-steel autoclave and maintained at 150 ^{o}C for 3-4 h. Finally, the product was harvested by centrifugation, washed by ethanol, followed by drying under dynamic vacuum at room temperature.

Synthesis of MTV-MIL-100(Ti, Mn). A typical synthesis was carried out by dissolving 0.40 mmol of benzene-1,3,5-tricarboxylic acid and 0.60 mmol of $Mn(NO_3)_2 \cdot 4H_2O$ in a mixture of 60 mL of ethylene glycol and 10 mL of *N*,*N*-dimethylacetamide. 89.0 μL of TIPO was added under ultrasonication. After forming a clear solution, it was transferred into a 100 mL Teflon-lined stainless-steel autoclave and maintained at 150 ^{o}C for 3-4 h. Finally, the product was harvested by centrifugation, washed by ethanol, followed by drying under dynamic vacuum at room temperature.

Synthesis of MTV-MIL-100(Ti, Fe). A typical synthesis was carried out by dissolving 0.40 mmol of benzene-1,3,5-tricarboxylic acid and 0.60 mmol of $FeCl_2 \cdot 4H_2O$ in a mixture of 60 mL of ethylene glycol and 10 mL of *N*,*N*-dimethylacetamide. 89.0 μL of TIPO was added under ultrasonication. After forming a clear solution, it was transferred into a 100 mL Teflon-lined stainless-steel autoclave and maintained at 150 ^{o}C for 3-4 h. Finally, the product was harvested by centrifugation, washed by ethanol, followed by drying under dynamic vacuum at room temperature.

Synthesis of MIL-125(Ti). In a typical synthesis, 667 uL of TIPO (2.26 mmol) and 750 mg of terephthalic acid (4.5 μmol) were dissolved in a mixture of 48 mL of *N*,*N*-dimethylformamide and 12 mL of methanol. This mixture was then heated in solvothermal conditions at 150 °C for 3 days. A white powder is obtained by centrifugation and dry under dynamic vacuum at room temperature.

Section 3. Synthetic route of AB_2OX_3 clusters and MTV-MIL-100(A,B).

MTV-MIL-100(A,B) has a general formula of $[AB_2(\mu_3\text{-}O)X_3(BTC)_2]$, where A and B are the desired metal cations and X sites can either be neutral H_2O molecules or OH^- anions to balance the charge. In the synthesis, DMA can convert to its corresponding amines at the high temperature, and it synergistically control the reaction kinetics and crystal growth along with EG. Due to charge balance, the total positive charges carried by A and B cations are limited. With the high excess of EG, the synthesis is carried out in a slightly acidic environment. Thus, the X sites prefer to be neutral H_2O molecules rather than OH^- anions. As a result, during the crystal growth process, A and B cations prefers to form the clusters that can minimize the total charge. Keeping this in mind, we successfully obtained a series of MTV-MIL-100(Ti,B), where the molar ratio of Ti/B (M = Co, Ni, Fe, Mn) = 0.5 (Figures S1 and S2). Altering the starting ratio of Ti/Co (0.05-0.5) resulted in the same compound with a fixed ratio of Ti/Co = 0.5 in the obtained crystals (Figures S3 and S4). We applied a similar approach for the single-metal component of Ti and Ni. When Ti and Ni occupy both A and B sites, it results in a total charge number of 12 and 6, respectively. Because the total charge number is out of the possible range where X sites can balance the charges, the resulting products are amorphous (Figure S5).

Section 4. XANES and XAS analysis.

The XANES and XAS measurements were performed at the Ti-K edge in transmission mode (4966 eV) at P65, Petra-III, DESY[1]. All the samples were measured as pellets and were optimized for unit step jump using cellulose. The data processing of pre-edge and post-edge background removal were performed using customized in-house developed software package, which were followed by Fourier transformation and data fitting. The k^3-weighted EXAFS oscillations were analysed by nonlinear least-squares refinement of the data. The parameters, mean number of neighbor distances (***N***), mean distances (***d***), Debye-Waller coefficients ($\boldsymbol{\sigma^2}$), and Many-body amplitude reduction factor ($\boldsymbol{S_0^2}$) were refined. The theoretical phases and amplitudes were calculated using *FEFF*[2,3]. All fitting parameters were varied during the fit, except ΔE_0, which was kept constrained to the value of the first Ti-O shell for all the shells. Besides, the value of $\boldsymbol{S_0^2}$(0.8) was estimated by fitting the EXAFS spectra on TiO_2 reference and kept fixed during the fit. With this approach, a slight deviation in the CN from the actual value is expected due to its correlation with $\boldsymbol{\sigma}$ and structural or compositional disorder. In addition to the Fourier transform structure modelling, a Cauchy wavelet transform (CCWT) analysis was performed. The contour plots of CCWT were generated using the computation MATLAB code by Muñoz et al. [4,5].

The XANES spectra of the MTV-MIL-100(Ti,Co) and the bulk Ti-metal, anatase and rutile references are shown in Figure 1e. The pre-edge peaks are typically associated with the coordination geometry of Ti atoms, e.g. the octahedral coordination geometry, where the 1s to 3d transition hybridized with 4p along with the 4p–4s hybridization [6,7]. Therefore, different orientation and local symmetry of Ti atoms lead to changes in intensity and energy level of the peaks. The pre-edge peaks of anatase and rutile (Figure S7) show the t_{2g} low energy states (A1,

A2 and A3) split from Ti 3d and an e_g high energy state (B). In contrast, the pre-edge peaks of MTV-MIL-100(Ti,Co) exhibits an asymmetric shoulder peak at A1 region. In addition to the absence of the high energy e_g peak B, it thus suggests a distorted octahedral coordination geometry[8–10] of the Ti atoms in the AB_2OX_3 clusters as compared to TiO_2. This distortion can be resulted from the removal of the terminal bonded solvent molecules to the Ti atoms, as well as the presence of neighbouring heteroatoms.

A simplified EXAFS equation can be expressed as,

$$\chi(k) = \sum_j \frac{N_j S_o^2 f_j(k) e^{\frac{-2R_j}{\lambda}} e^{-2k^2 \sigma_j{}^2}}{kR^2} \sin[2kR_j + \delta_j(k)]$$

where N is the mean number of neighbour distances, R is the mean distances, σ^2 is the Debye-Waller coefficients, $f(k)$ is the many-body scattering amplitude, $\delta(k)$ is the mean-square disorder of neighbour distance, S_0^2 is the many body amplitude reduction factor, and λ is the mean free path. The isolation of EXAFS signal ($\chi(k)$) is a function of scattering amplitude $f(k)$ and phase shift $\delta(k)$, which are specific to atomic mass (Z). Therefore, depending on the absorber-backscatterer pair, the $f(k)$ is localized within a specific k-range within k-space. Post the normalization and isolation of $\chi(k)$, the conventional EXAFS modelling involves Fourier transformation of the $\chi(k)$ to FT magnitude in the R-space. The type of atomic species can be interpreted based on the modelling of the FT magnitude. This is based on the deconvolution of frequency in the k-space signal. However, the nature of atomic species when located at the same radial distance cannot be accurately distinguished with this approach. Thus, an alternative approach such as Cauchy wavelet transform (CCWT), which provides simultaneous resolution in k and R-spaces is insightful. This provides qualitative and more accurate information on the type of atomic species. This scattering amplitude shifts to a higher k-range with increasing Z, which results in localization of atomic species in a specific k-range.

This phenomenon is shown in Figure S12 from the theoretical $f(k)$ amplitudes calculated using *FEFF* [2,3]. The effective $f(k)$ of Ti-C, Ti-Co and Ti-Ti with respect to a central absorber Ti show that the Ti-Co scattering path shows a maximum at relatively higher k-ranges (Figure S12) when compared to other species (Ti-C, Ti-O and Ti-Ti). The isolation of the maximal amplitudes based on Z could provide insight on the presence of a heterogeneous cluster (i.e. Co-Ti-Co or Co-Co-Ti), which would lead to an asymmetric wavelet at the same radial distance. Hence, CCWT analysis was used to examine the presence of a heterogeneous cluster.

The maximal amplitudes of Ti-Ti atoms in the bulk Ti metal is shown in Figure S13. The maximal backscattering amplitude spread of the Ti-Ti clusters at various radial distances was observed ~7-8 $Å^{-1}$. At the Ti-K edge, the first shell amplitude maximum for MTV-MIL-100(Ti,Co) localized at k ~6 $Å^{-1}$ and ΔR = (1.5–2) Å (Figure S14), which suggests the presence of oxygen in the first shell. Furthermore, the second shell has a wavelet maximum at k ~7.5 $Å^{-1}$ and ΔR = (2.5–3.5) Å with a distorted maximum suggests the presence of a heteroatoms in the cluster. In addition, the presence of a lobe at ΔR = (3–3.5) Å and low k ranges indicates the presence of light atoms

such as C atoms which link to the clusters in the higher coordination shell. However, CCWT analysis is insufficient to identify the type of cluster Co-Co-Ti/Co-Ti-Ti. A detailed FT modelling might also complement the information.

Based on the insights from the CCWT analysis and XANES, a FT analysis was performed. The presence of peaks at ~1.3 Å and ~1.8 Å suggest the presence of two species in the first shell. This could be assigned to ligands which are bonded to the clusters. The FT model indicate the presence of chemically bonded oxygen and ligands (Figure S31 and Table S4). However, data quality of the FT model is insufficient to deduce coordination in the higher shells.

Section 5. Computational study.

Density functional theory (DFT) geometry optimizations were carried out using the M06-L density functional[11] and the Gaussian 09[12] software package. The def2-SVP basis set was employed for H, C, and O atoms, and the def2-TZVPP basis set[13,14] was employed for the Co and Ti atoms. The spin states ladder was analyzed with the M06-L functional, while the electronic structure analysis and time-dependent density functional theory (TDDFT) calculations were carried out using the B3LYP hybrid density functional[15] and the HSE06 hybrid density functional.[16] For the electronic structure and TDDFT analysis, we included the solvent effect (water) using the polarizable continuum model (PCM).[17,18] An ultrafine grid was used for performing numerical integrations. All calculations have been carried out in the unrestricted formalism.

We first analyzed the spin states ladder, and from XPS analysis we know that there is a Co(III) and a Co(II) in the cluster, and thus there are seven unpaired electrons at maximum. For this analysis we modeled the MOF using a cluster approach truncating the linkers to formate groups (Figure S24 and Table S5), because the spin does not arise from the linkers (which are organic closed-shell molecules). One of the ligand on the Co(III) has been modeled with an OH^- group and the other ligands (apart from the formate groups) have been modeled with H_2O. In this way, the cluster is overall neutral. We then analyzed all the possible spin states arising from seven unpaired electrons, optimizing the geometry for each spin state, and the relative energies are shown in Table S2. We also modeled the doublet broken symmetry (BS) solution, in which there are four α unpaired electrons on Co(III) and three β unpaired electrons on Co(II). From Table S2 we can see that the octet is the most stable spin state, with the doublet (broken symmetry) spin state lying close to the octet. We therefore carried out the computational modeling of the electronic structure within the octet spin state.

For the study of molecular orbitals diagram, we use a cluster model that includes the heterometallic core, four linkers truncated as formate groups and two full linkers in which the COO^- groups are capped with hydrogen atoms (Figure S11). We looked at the molecular orbitals diagram and since we performed the calculations in an unrestricted formalism, we will have a set of α orbitals and a set of β orbitals. From Figure 3, we notice that the HOMO is mostly a *d* orbital

with β spin localized on Co(II). We set the energy of the HOMO as zero, and we report the energy of the other orbitals as relative energies. The LUMO is calculated to consist a *d* orbital (with β spin) localized on Co(III), located at 2.54 eV. At higher energy, we found an empty orbital (α and β have the same energy) at 3.51 eV, which is a mixture of a *d* orbital of Ti(IV) and a *d* orbital of Co(III). At 3.68 eV, a *d* orbital almost completely localized on Ti was found. Moreover, the first occupied orbital of the organic linker is located at -1.03 eV (Figure 3). Results with the HSE06 functional yield the same orbital pictures, with transition energies within 0.2 eV with respect to the B3LYP results.

Another way to produce a hole and an electron (spatially separated) is through a local excitation followed by a spatial splitting of the charges. This mechanism has been invoked, for example, in cerium photocatalytic MOFs[19]. We therefore analyzed the local excitation within BTC using TDDFT for a linker model only, as shown in Figure S25, left. We found that the first bright excitation corresponds to a Π-Π* transition, and the natural transition orbitals (NTOs) are shown in Figure S25, right. The oscillator strength for this transition is 0.462 (bright excitation), but it is located at 5.77 eV, much higher than the visible light range used experimentally. We can therefore rule out a mechanism involving a local excitation within the organic linker.

Section 6. Photocatalytic H_2 production.

Pt was loaded to MTV-MIL-100(Ti, Co), MIL-125(Ti), and P25 as a co-catalyst by a double solvents approach[20]. Typically, 200 mg of MTV-MIL-100(Ti, Co) was added to 40 mL of dry hexane as hydrophobic solvent, and then sonicated for 20 min and stirred for 2 h. After that, 0.205 mL of 10 mM H_2PtCl_6 as hydrophilic solvent was added dropwise (with 20 min) under vigorous stirring. After continuously stirred for 2 h, the powder was filtrated and dried in air at room temperature. As-synthesized sample was then moved to tube furnace and treated in a stream of H_2/He at 200 ^oC for 5 h to obtain loaded Pt inside MTV-MIL-100(Ti, Co).

Photocatalytic performance of the catalysts for hydrolysis of ammonia borane (AB) was evaluated under the irradiation of visible light (360-780 nm) at room temperature. The light power for the photocatalysis here is fixed to 300 J s^{-1}. For estimation of quantum efficiency (QE), the photocatalytic hydrolysis of AB was measured under the irradiation of monochromatic visible light (520 nm). Typically, 2.4 mg of the catalysts was dispersed in 3.0 mL of H_2O and then sonicated for 10 min to obtain a uniformly dispersed solution. Before the solution test, the catalyst was first irradiated under the visible light for 20 min to activate the catalysts. Then, photocatalytic hydrolysis reaction of AB was stirred rapidly when 4.0 mL of 5.0 mg mL^{-1} AB was injected. The visible light was kept turning on during the photocatalysis. The produced gas was collected by a classic H_2O-displacement method[21,22]. All photocatalytic tests were repeated for five times, and for the estimation of QE, they are repeated for three times. The deviations among each test are less than 5%. The reaction kinetics was investigated by varying test temperatures at 298, 303, 308 and 313 K. For the durability measurements, the hydrolysis of AB was repeated five times under the

irradiation of visible light by adding another equivalent of AB (5.0 mg mL^{-1}, 4.0 mL) into the reaction mixture after complete conversion of AB at previous cycle.

The activation energy (E_a) was calculated by Arrhenius equation[23] as,

$$\ln(\mathrm{TOF}) = \ln A - E_a/RT \qquad (1)$$

where *TOF* is the reaction rate, *A* is the pre-exponential factor, *R* is the universal gas constant, and *T* is the testing temperature.

The QE was calculated as,

$$QE = \frac{2\times M\times N_A\times h\times c}{S\times P\times T\times \lambda} \qquad (2)^{24}$$

where *M* is the yield of H_2, N_A is the Avogadro constant (6.02×10^{23} mol^{-1}), *h* is the Planck constant (6.626×10^{-34} J s^{-1}), *c* is the speed of light in vacuum (3×10^8 m s^{-1}), *S* is irradiation area; *P* is the intensity of irradiation light, *T* is the photoreaction time, and λ is the wavelength of the monochromatic light.

The space time yield (STY) was calculated as,

$$STY = \frac{\text{Quantum yield (molecules per photon)}}{\text{Photocatalyst mass (mg)}} \qquad (3)^{25}$$

The Figure of Merit (FOM) was calculated as following, with the adaption of FOM for photocatalytic degradation of pollutants[26].

$$FOM = \frac{\text{Conversion efficiency (\%)}}{\text{Catalyst mass (mg)}\times Initial\ AB\ concentration\ (ppm)\times Irration\ time\ (min)\times Applied\ power\ (W)} \qquad (4)$$

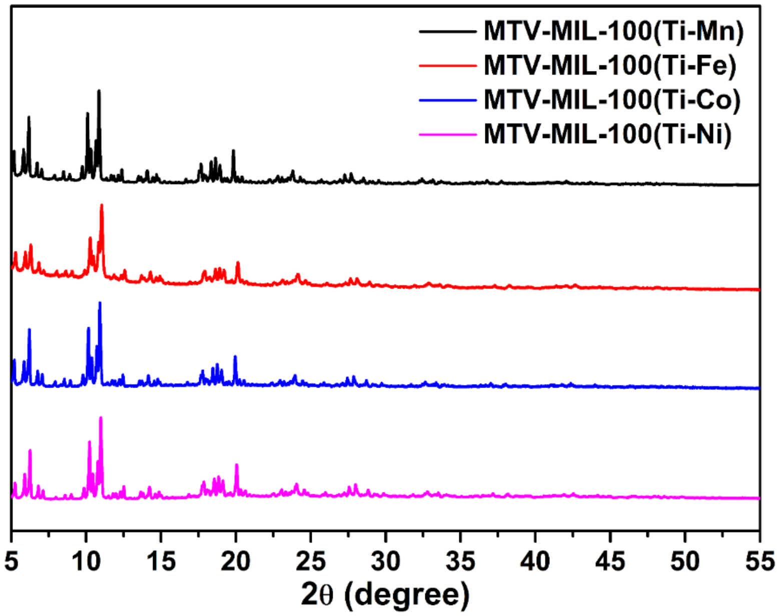

Figure S1. PXRD patterns of MTV-MIL-100(A,B). A = Ti; B = Co, Ni, Fe, Mn.

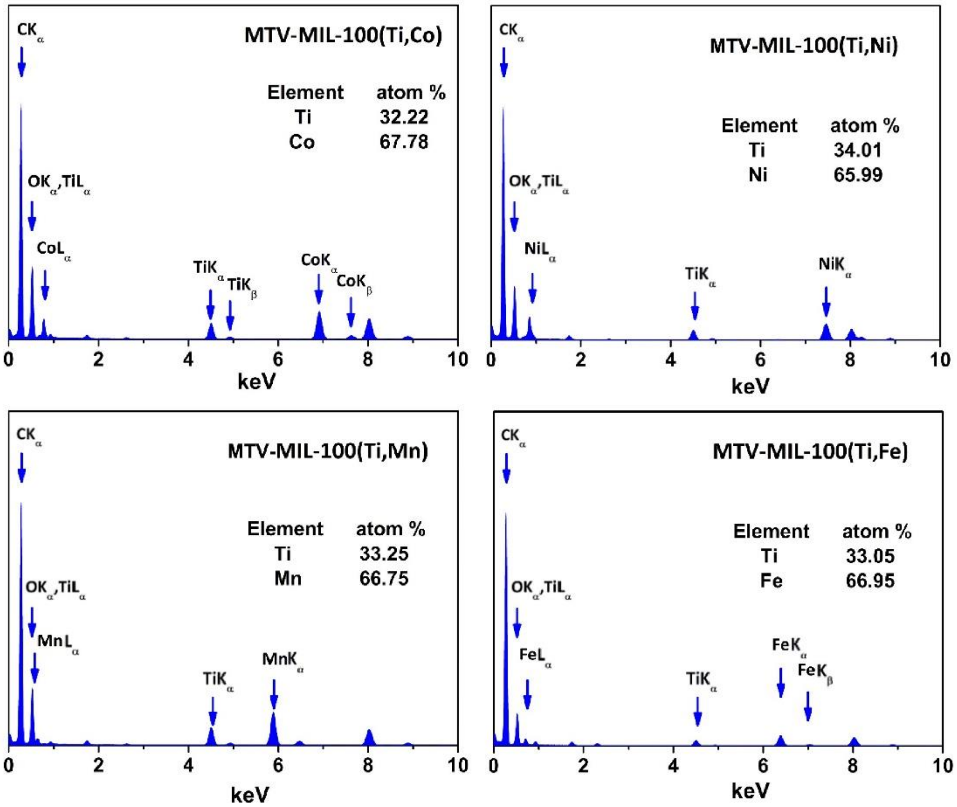

Figure S2. EDS spectra of MTV-MIL-100(A,B), showing the molar ratio of Ti/B = 0.5. A = Ti; B = Co, Ni , Fe, and Mn.

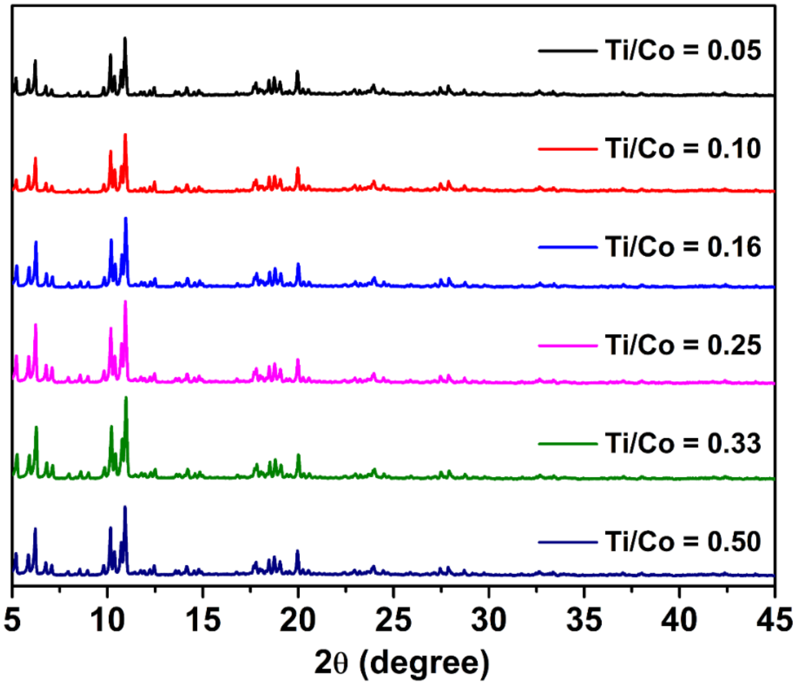

Figure S3. PXRD patterns of MTV-MIL-100(Ti,Co) synthesized under different starting Ti/Co ratios.

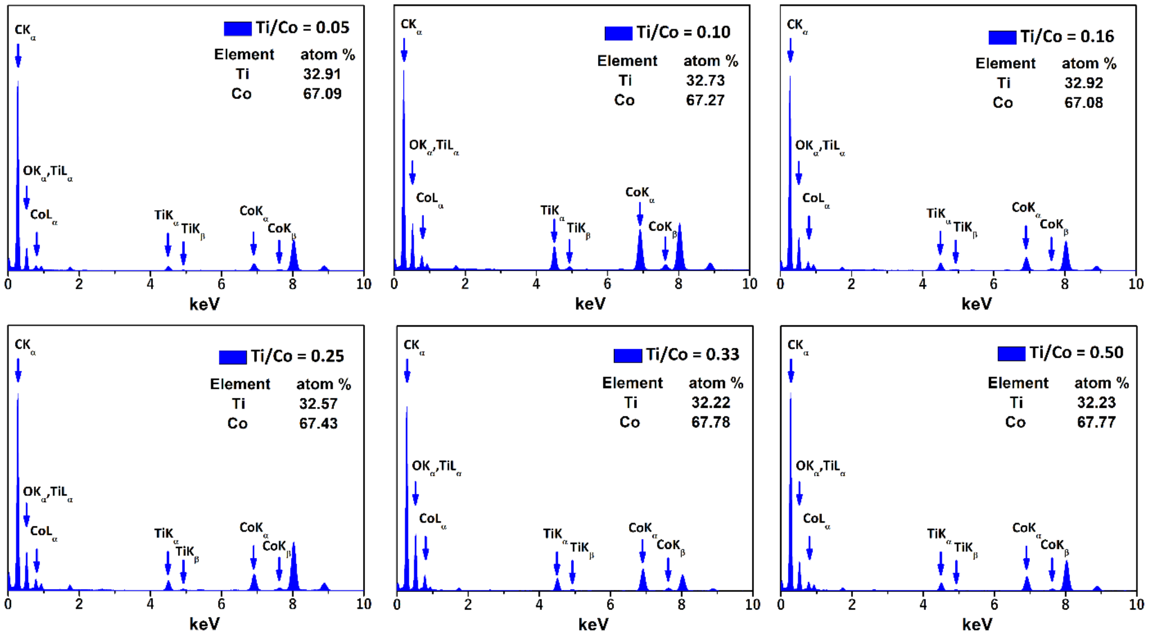

Figure S4. EDS spectra of MTV-MIL-100(Ti,Co) synthesized under different starting Ti/Co ratios from 0.05 to 0.5. Although started from a different Ti/Co ratio, the ratio of Ti/Co = 0.5 in the final crystals was obtained.

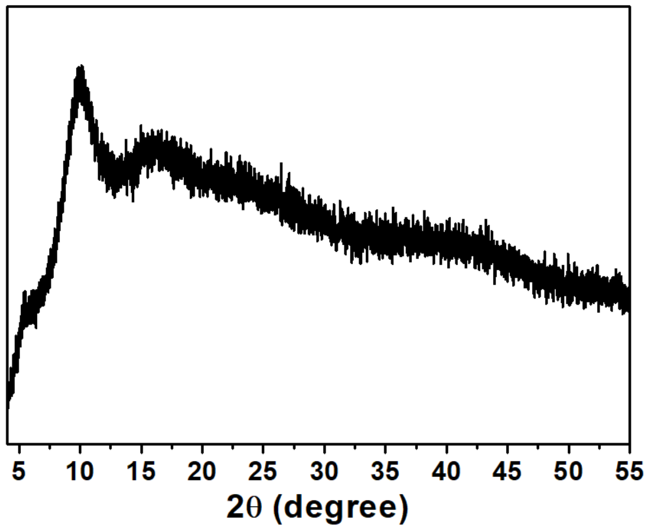

Figure S5. PXRD pattern of the resulting products from single metal component of Co. No crystal formed with similar experimental conditions with a single metal component of Ti.

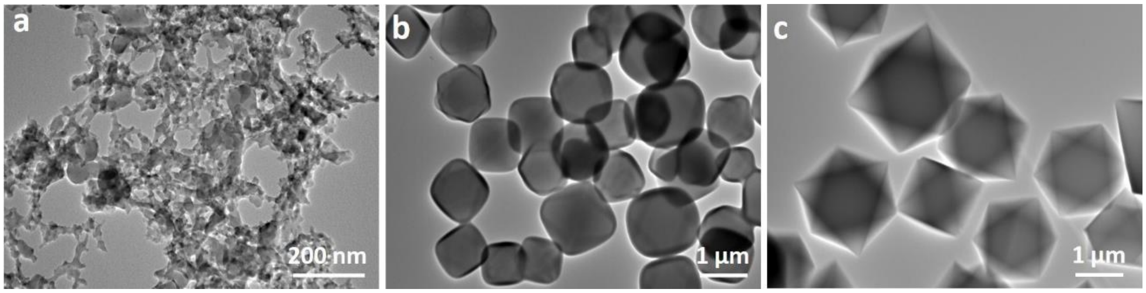

Figure S6. TEM images of MOFs synthesized with volume ratios of EG/DMA: (a) 0 : 70, (b) 20 : 50, (c) 60 : 10.

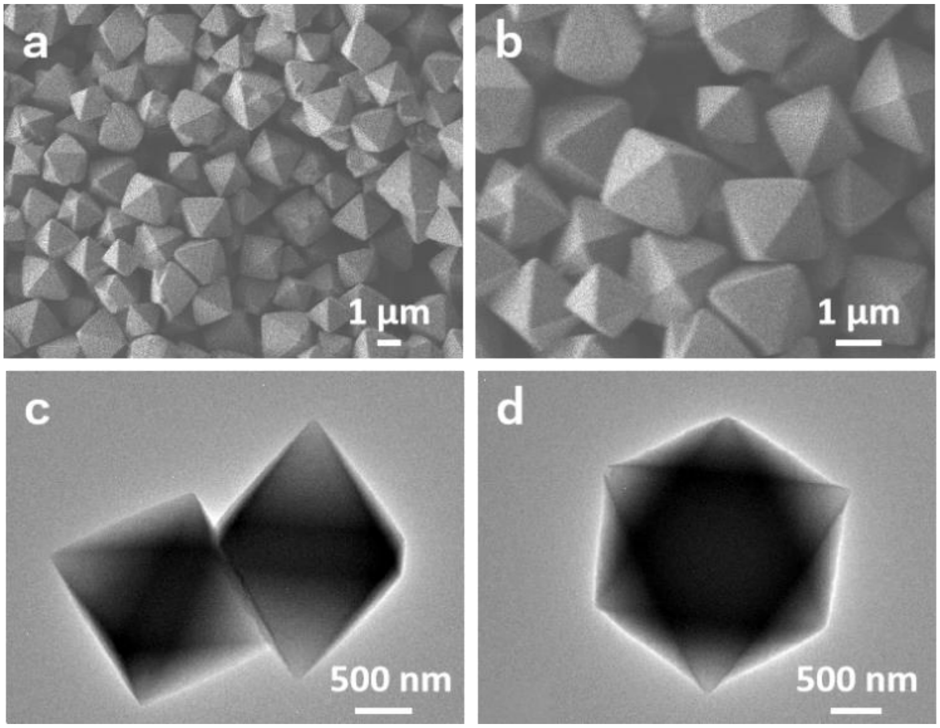

Figure S7. SEM (a,b) and TEM (c,d) images of MTV-MIL-100(Ti,Co), showing an octahedral morphology.

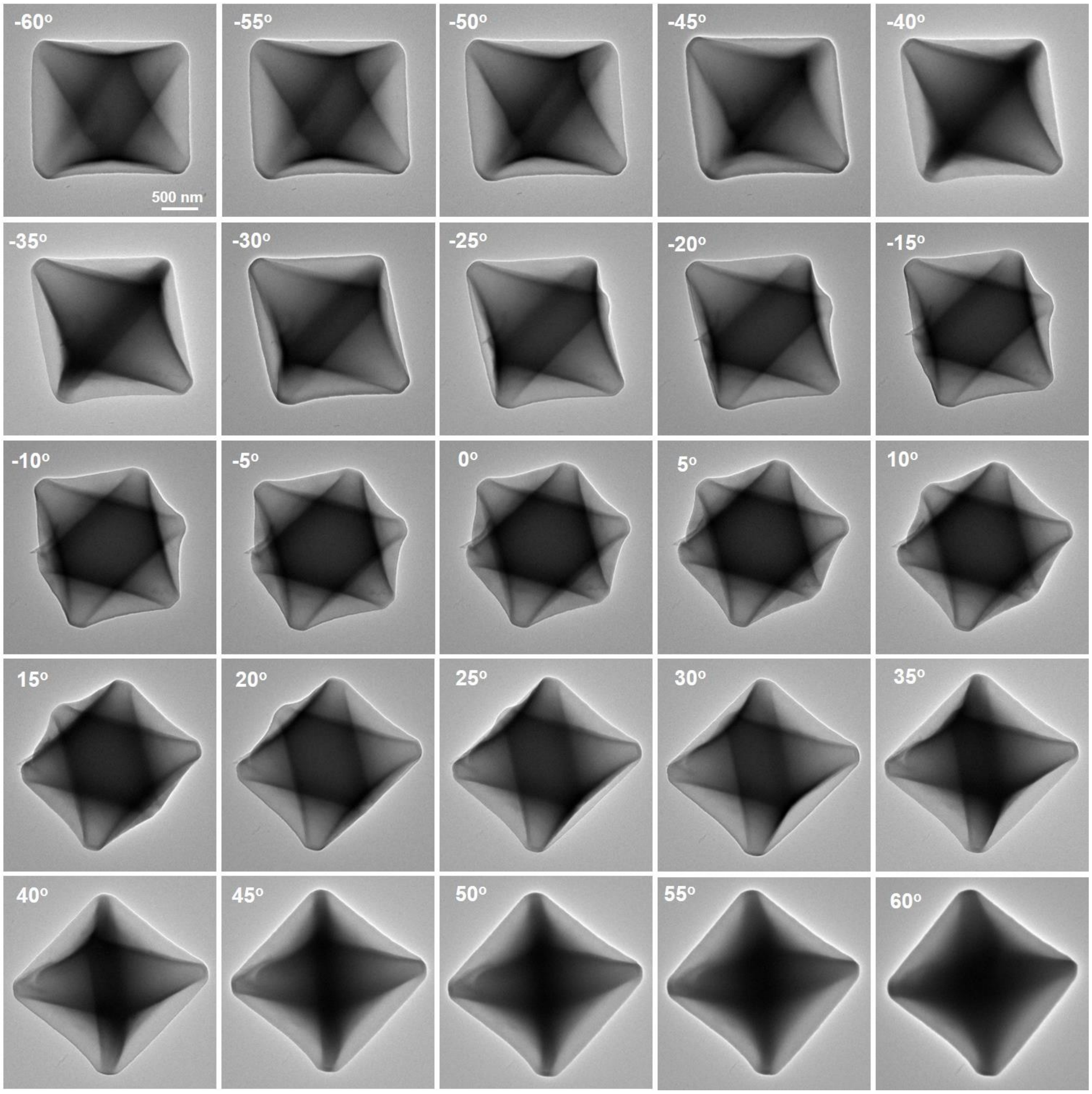

Figure S8. TEM images of MTV-MIL-100(Ti,Co) collected from different observation angles, indicating an octahedral morphology.

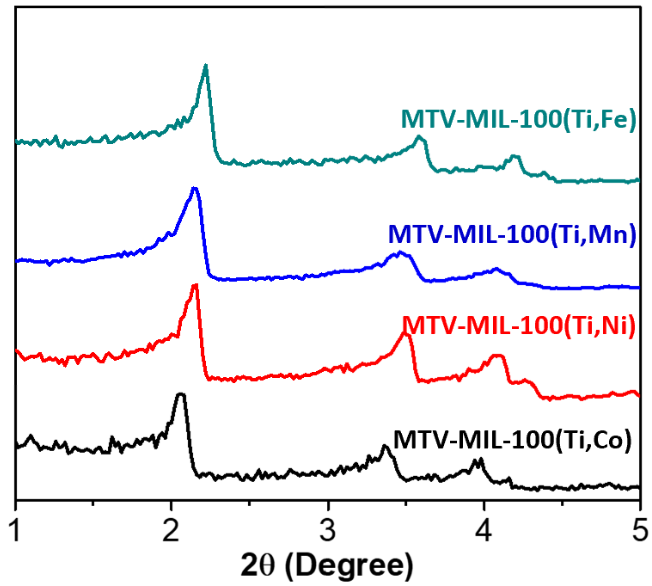

Figure S9. Small-angle XRD patterns of the MTV-MIL-100(Ti,B).

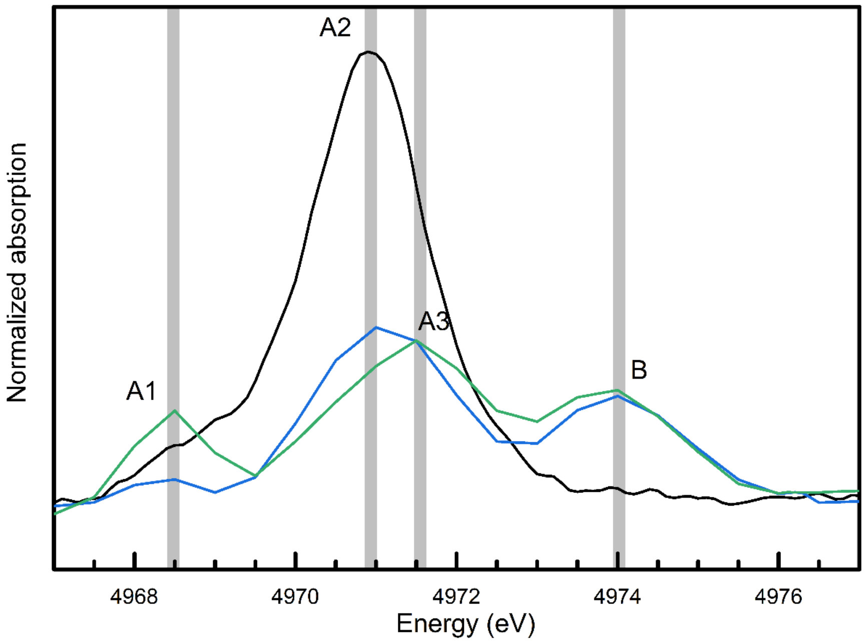

Figure S10. The background subtracted pre-edge XANES spectra of the MTV-MIL-100(Ti,Co) (black curve) comparing with rutile (blue curve) and anatase (green curve) references.

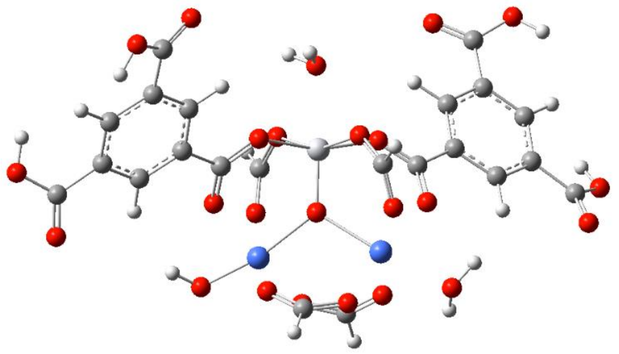

Figure S11. Ball and stick representation of the DFT calculated local structural model of MTV-MIL-100(Ti,Co) for the electronic structure analysis.

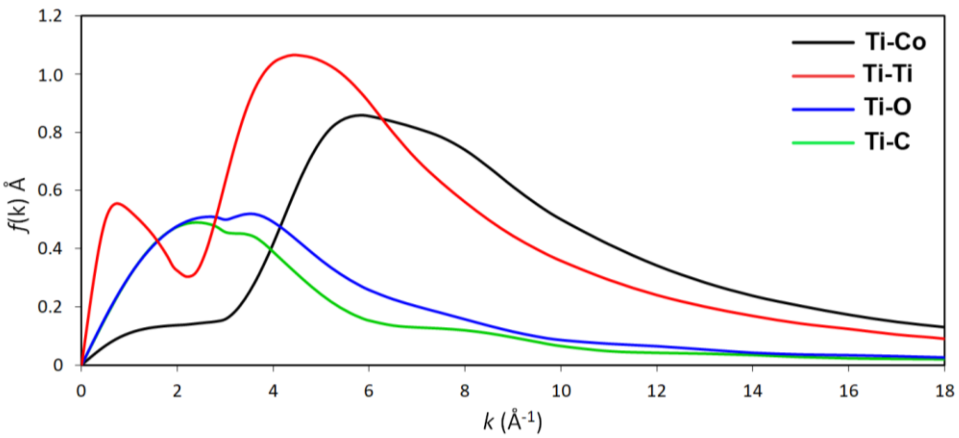

Figure S12. The scattering amplitude *f*(k) using Ti as the central absorber of the MTV-MIL-100(Ti,Co). The amplitudes was calculated using the *FEFF* package.

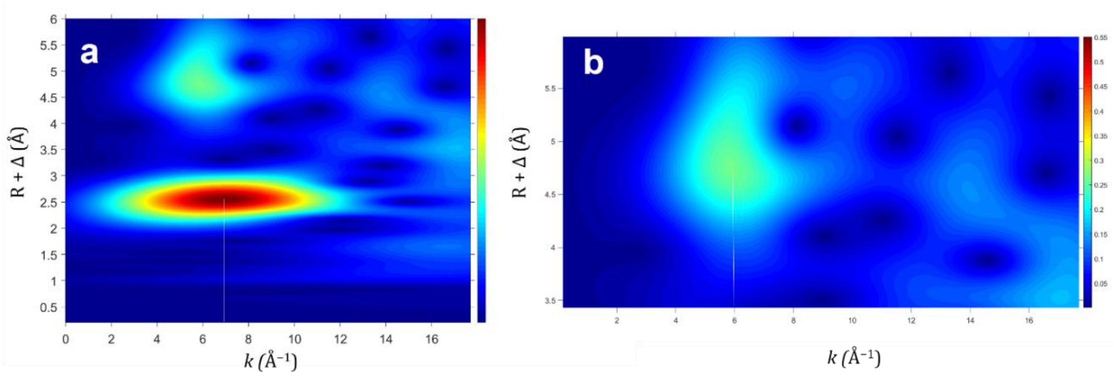

Figure S13. The CCWT of the bulk Ti metal at (a) Ti-K edges and (b) the second shell wavelet maxima.

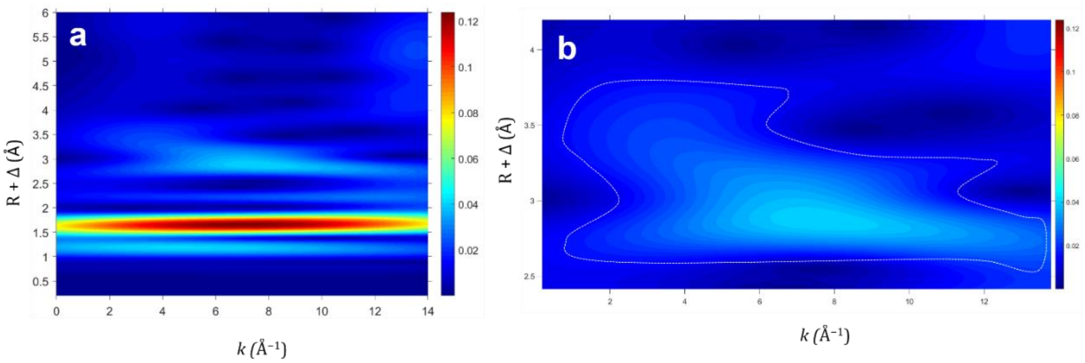

Figure S14. The CCWT of the MTV-MIL-100(Ti,Co) at (a) Ti-K edges and (b) the second shell wavelet maxima.

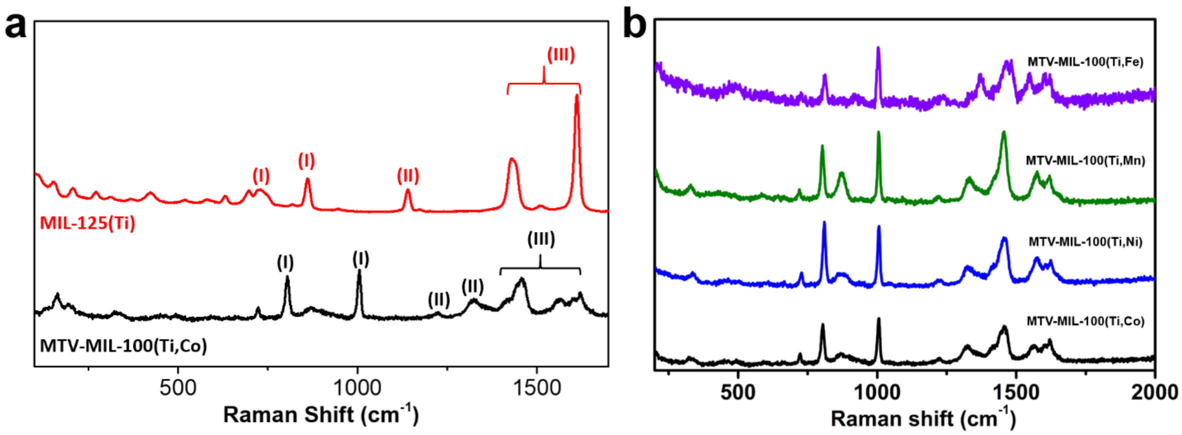

Figure S15. (a) Comparison of Raman spectra of MTV-MIL-100(Ti,Co) and MIL-125(Ti). (b) Raman spectra of MTV-MIL-100(A,B). A = Ti; B = Co, Ni, Mn, Fe. Compared to single component crystal (MIL-125(Ti)), Raman peaks are different for MTV-MIL-100(Ti,Co), indicating new crystalline structures. For peaks (I), two Raman signals correspond to the fingerprints of the trimesate linker and the aromatic ring peaks, respectively. The Raman peak (II) is assigned to the C-O-Ti-Co (C-O-Ti and C-O-Co) stretching of Ti/Co-trimesates. The peaks (III) are related to the H-O-H bonding vibrations coordinated within Ti/Co-trimesate.

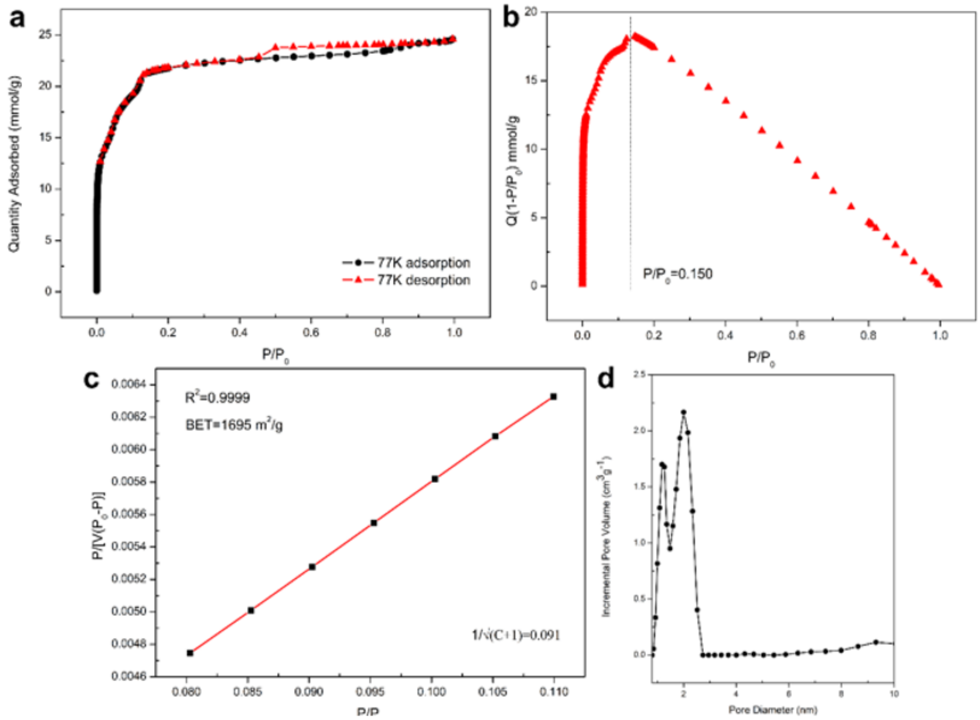

e

P/P$_0$ Range	0.0803-0.1100
Correction Coefficient	0.99999
C	121
Q_m (mmol g^{-1})	18.5
1√(C+1)	0.091
BET Surface Area (m^2 g^{-1})	1695

Figure S16. Porosity analysis of MTV-MIL-100(Ti,Co). (a-b) N_2 adsorption-desorption isotherms and the analysis of MTV-MIL-100(Ti,Co) at 77 K. (c) Plot of the linear region of the adsorption N_2 isotherm used for the BET equation. (d) Pore size distributions calculated by DFT. (e) Summary of parameters in the BET analysis.

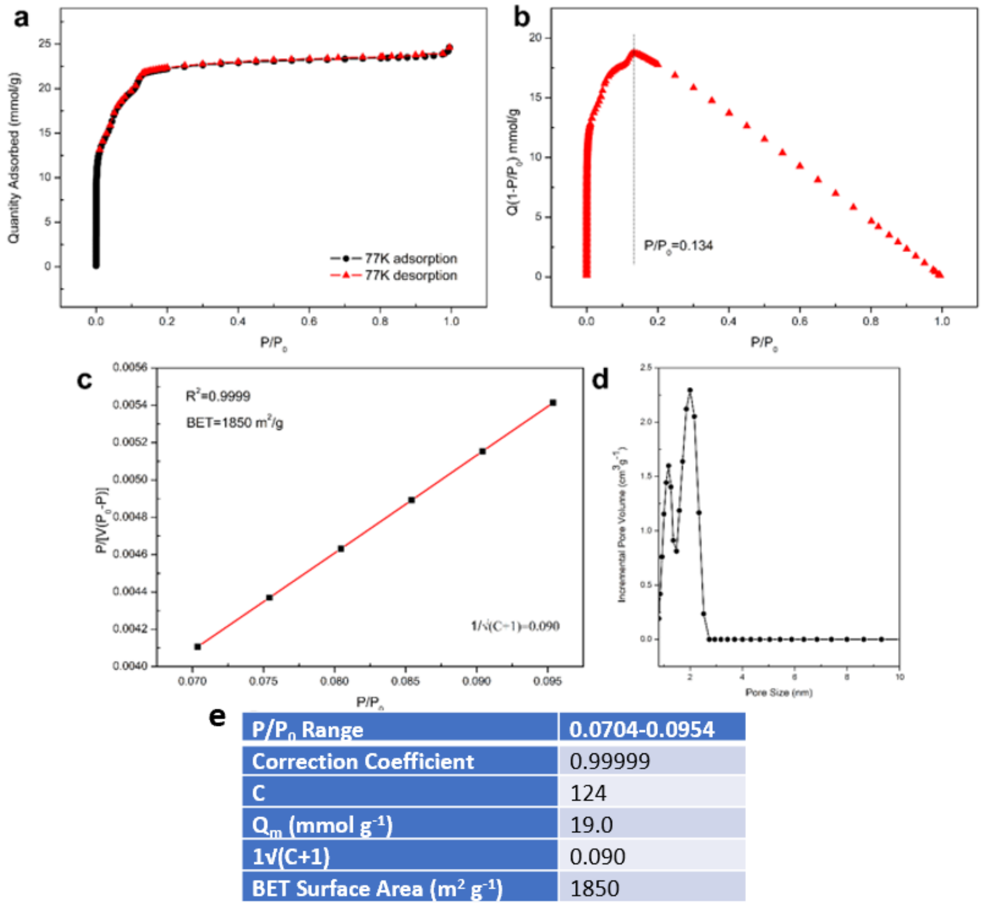

e

P/P_0 Range	0.0704-0.0954
Correction Coefficient	0.99999
C	124
Q_m (mmol g^{-1})	19.0
1√(C+1)	0.090
BET Surface Area (m^2 g^{-1})	1850

Figure S17. Porosity analysis of MTV-MIL-100(Ti,Ni). (a-b) N_2 adsorption-desorption isotherms and the analysis of MTV-MIL-100(Ti,Ni) at 77 PK. (c) Plot of the linear region of the adsorption N_2 isotherm used for the BET equation. (d) Pore size distributions calculated by DFT. (e) Summary of parameters in the BET analysis.

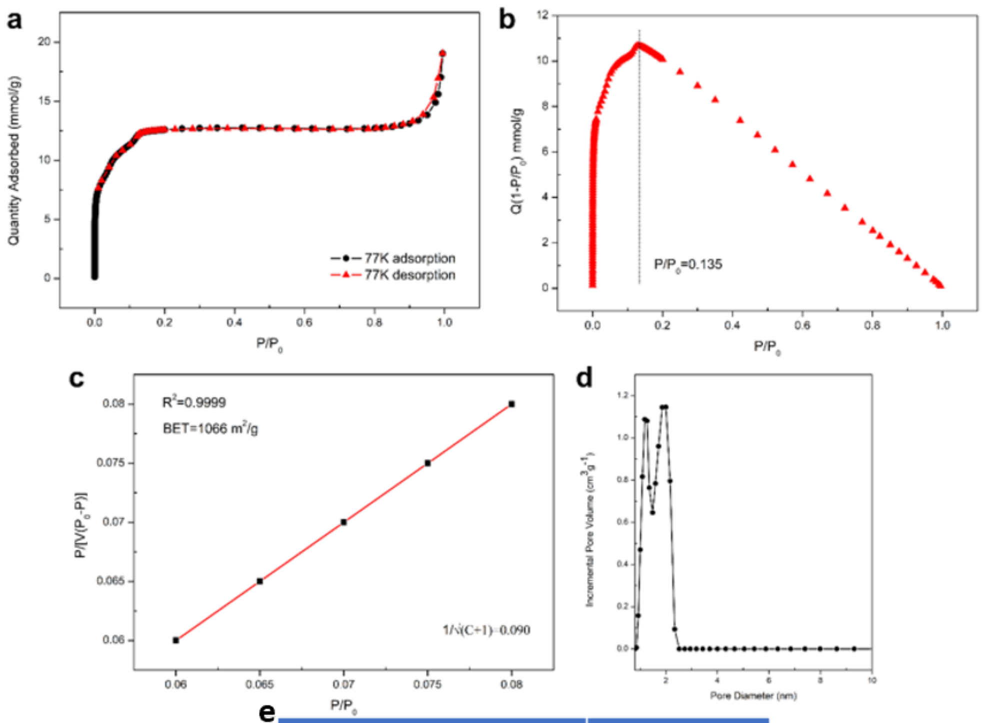

e

P/P_0 Range	0.0601-0.0801
Correction Coefficient	0.99999
C	124
Q_m (mmol g^{-1})	10.9
1√(C+1)	0.090
BET Surface Area (m^2 g^{-1})	1066

Figure S18. Porosity analysis of MTV-MIL-100(Ti,Fe). (a-b) N_2 adsorption-desorption isotherms and the analysis of MTV-MIL-100(Ti,Fe) at 77 K. (c) Plot of the linear region of the adsorption N2 isotherm used for the BET equation. (d) Pore size distributions calculated by DFT. (e) Summary of parameters in the BET analysis.

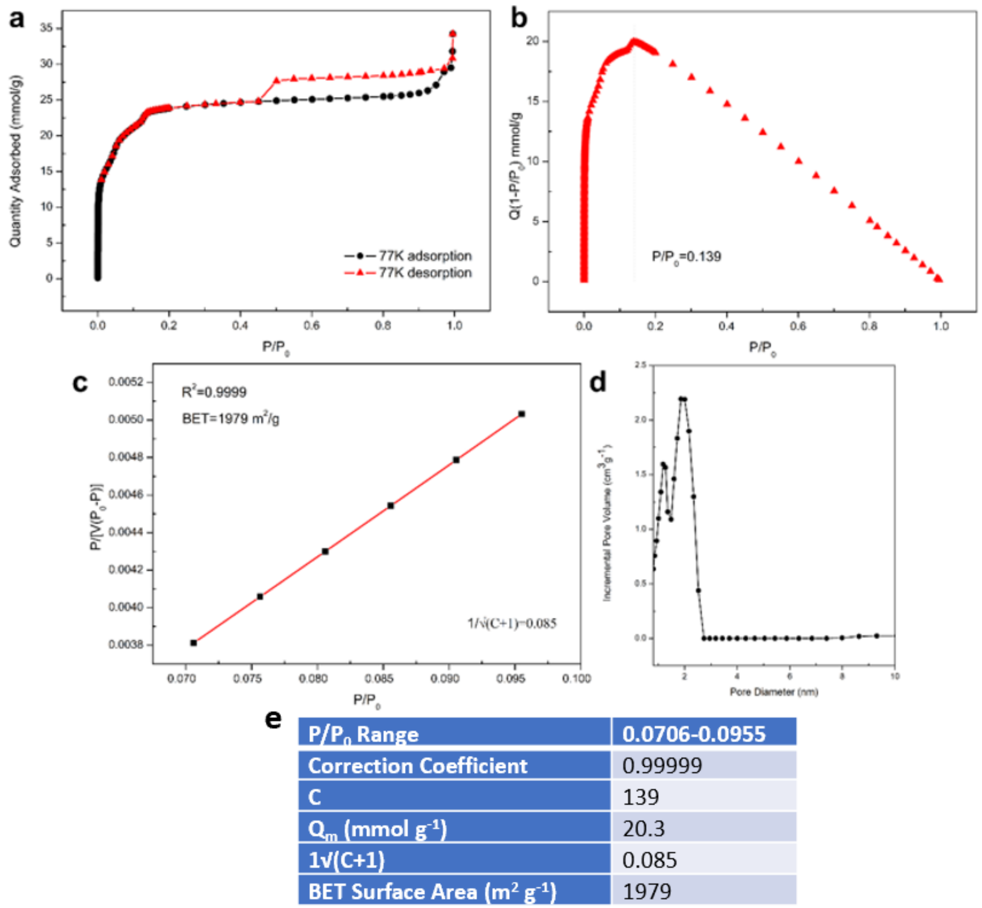

e

P/P_0 Range	0.0706-0.0955
Correction Coefficient	0.99999
C	139
Q_m (mmol g^{-1})	20.3
1√(C+1)	0.085
BET Surface Area (m^2 g^{-1})	1979

Figure S19. Porosity analysis of MTV-MIL-100(Ti,Mn). (a-b) N_2 adsorption-desorption isotherms and the analysis of MTV-MIL-100(Ti,Mn) at 77 K. (c) Plot of the linear region of the adsorption N2 isotherm used for the BET equation. (d) Pore size distributions calculated by DFT. (e) Summary of parameters in the BET analysis.

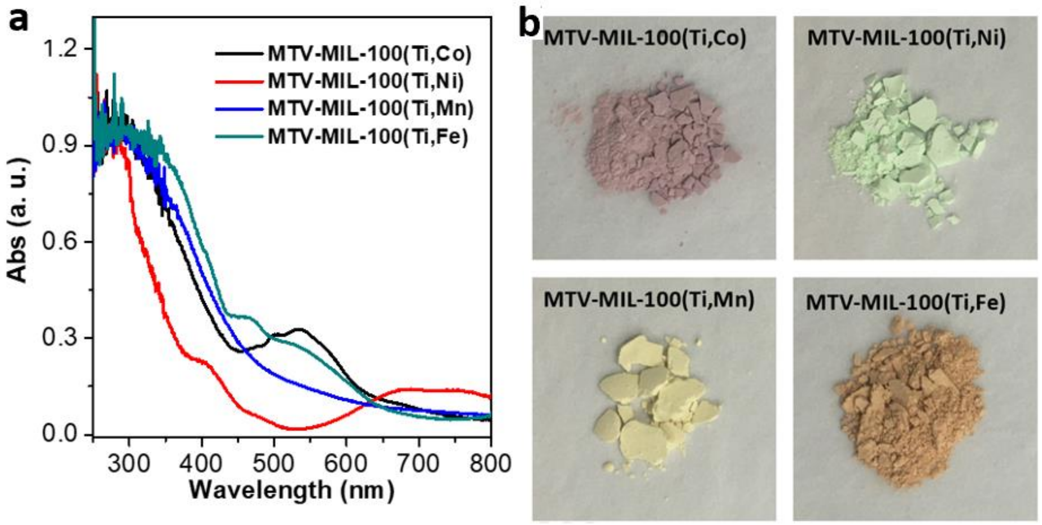

Figure S20. (a) UV-vis spectra and (b) corresponding photographs of the MTV-MIL-100(Ti,B). B = Co, Ni, Fe, Mn.

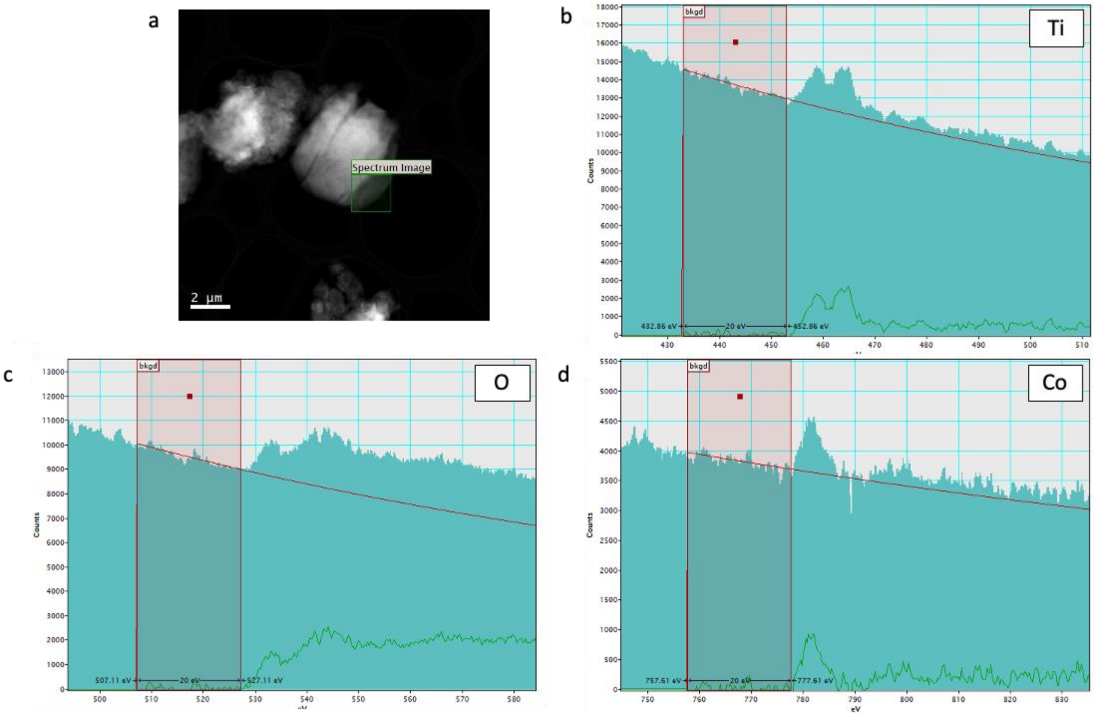

Figure S21. Electron Energy Loss Spectroscopy (EELS) data collected from a MTV-MIL-100(Ti,Co) crystal. (a) HAADF-STEM image of the studied crystal. (b-d) EEL spectra from showing the Ti, O and Co edges respectively after correction for energy shift and background removal.

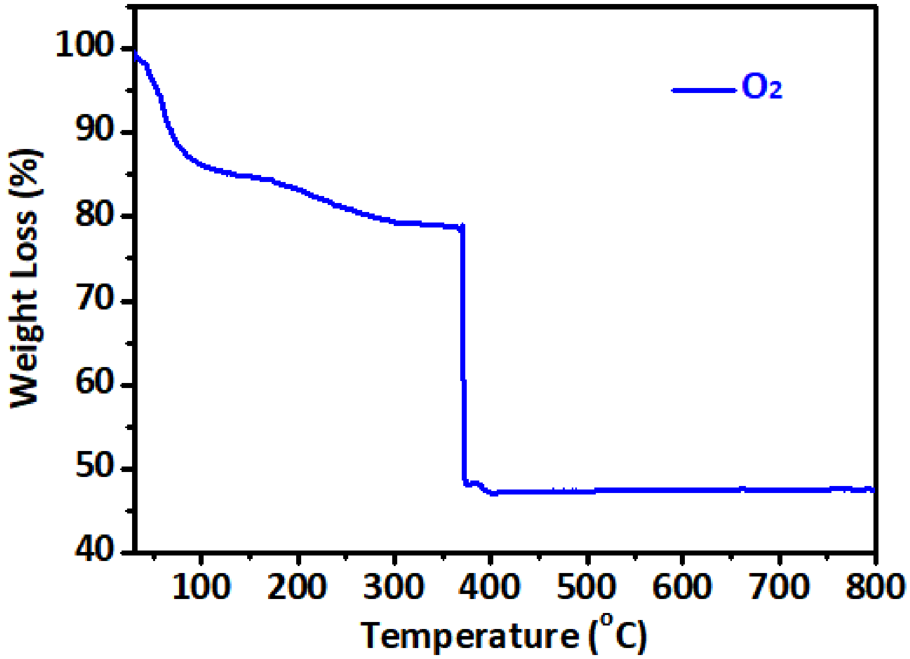

Figure S22. TGA of MTV-MIL-100(Ti,Co) under O_2 atmosphere.

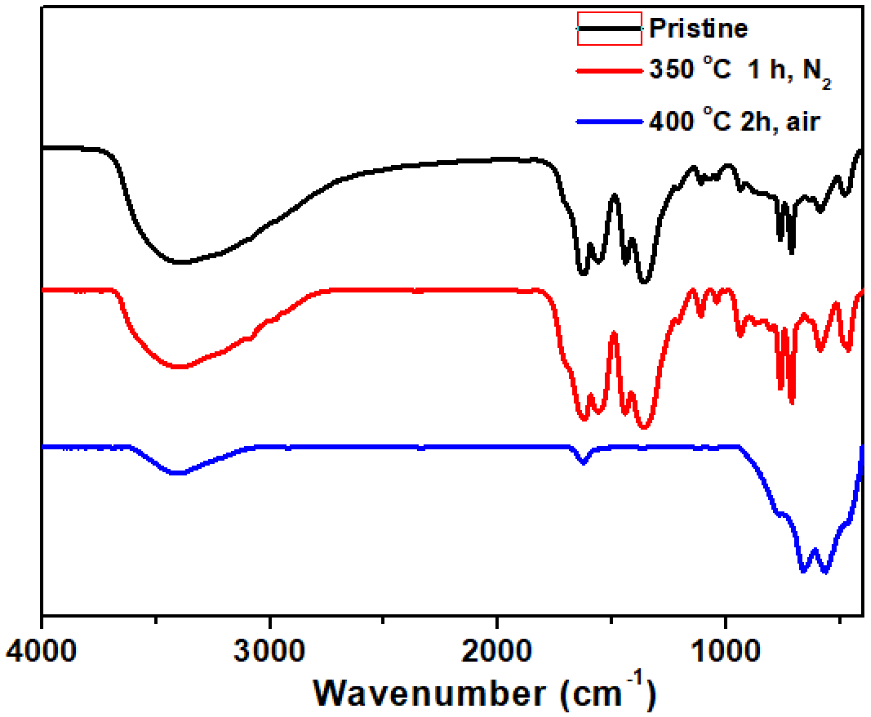

Figure S23. FT-IR spectra of the MTV-MIL-100(Ti,Co) with different thermal treatments.

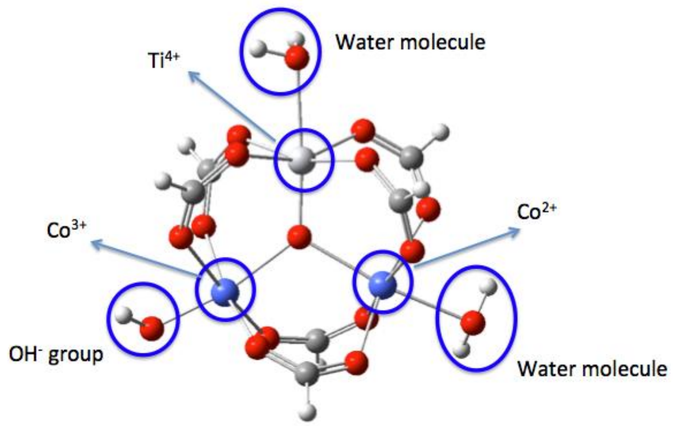

Figure S24. Ball and stick representation of the computational model used for spin states analysis.

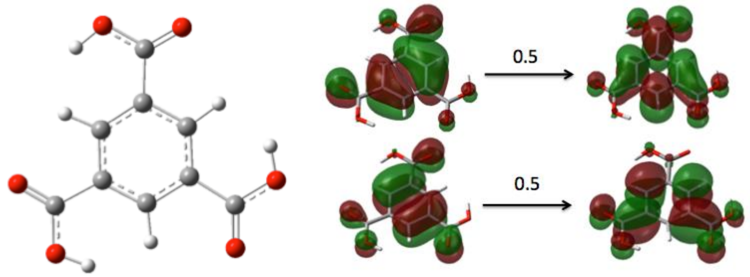

Figure S25. Ball and stick representation of the linker model for TDDFT (left) and NTOs for the first bright excited state of the linker (right).

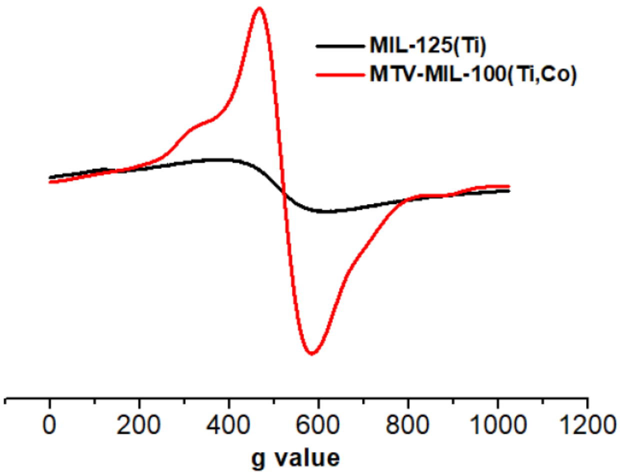

Figure S26. EPR spectra of the MTV-MIL-100(Ti,Co) and MIL-125(Ti).

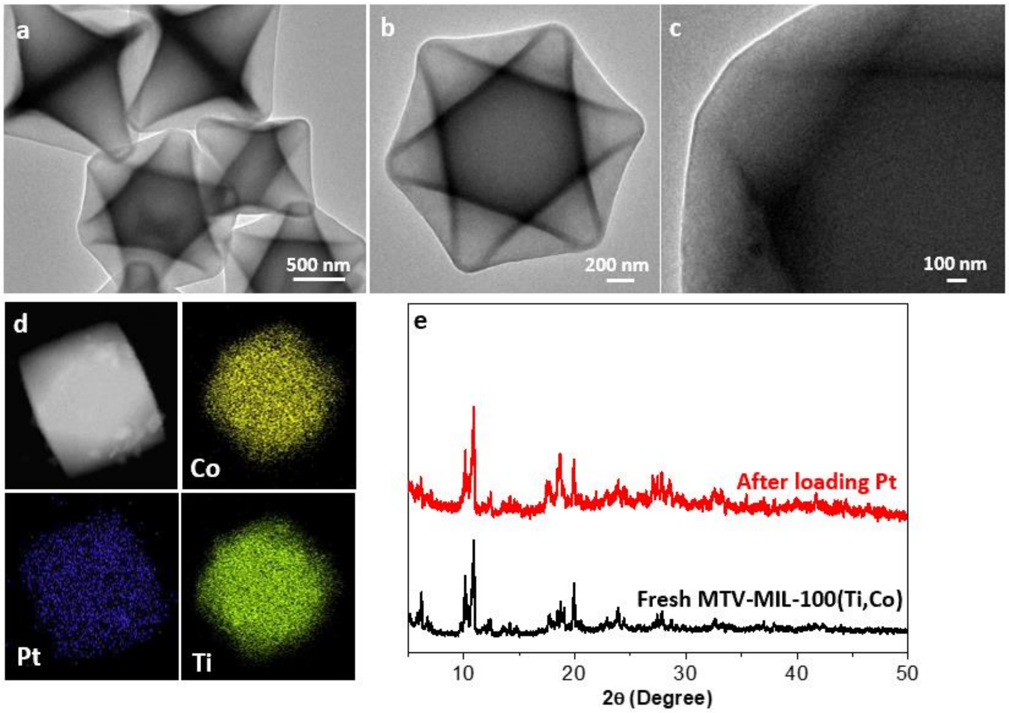

Figure S27. (a-c) TEM images and (d) STEM elemental maps of MTV-MIL-100(Ti,Co) loading with Pt nanoparticles. (e) PXRD patterns of MTV-MIL-100(Ti,Co) before and after loading with Pt nanoparticles.

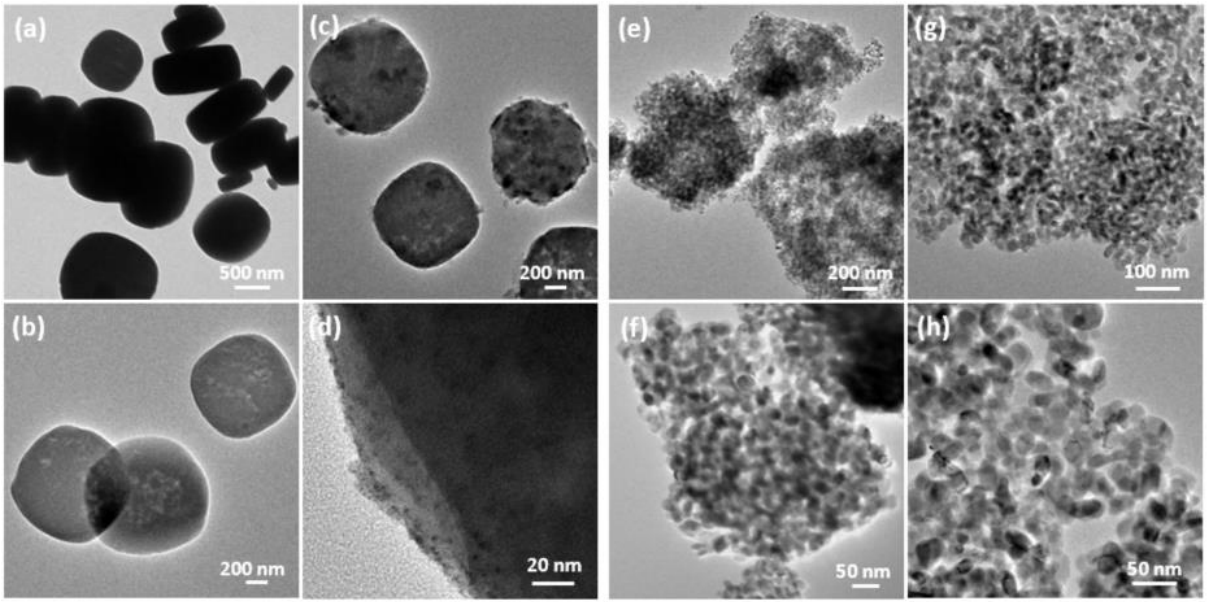

Figure S28. TEM images of (a-d) MIL-125(Ti) and (e-h) P25 (a,b,e,f) before and (c,d,g,h) after loading Pt nanoparticles.

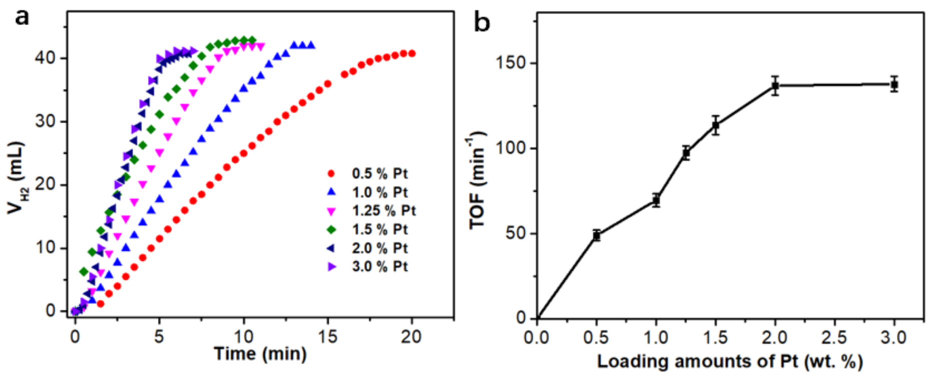

Figure S29. (a) Catalytic activities and (b) calculated TOF values of AB photolysis with different Pt loading amounts.

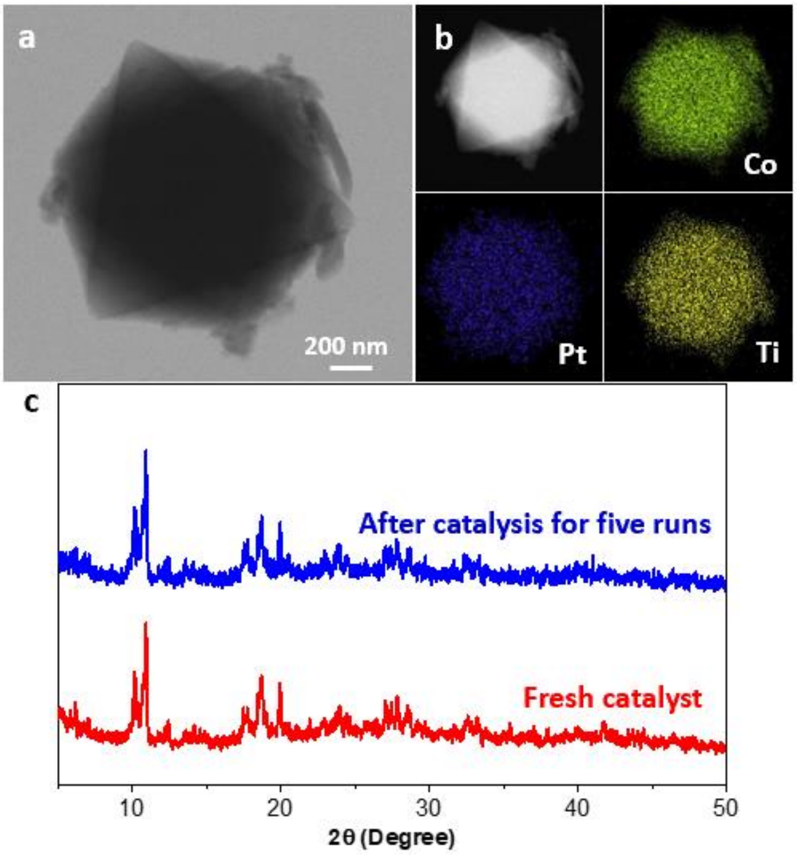

Figure S30. (a) TEM and (b) STEM elemental maps of MTV-MIL-100(Ti,Co) after catalysis for 5 runs. (c) PXRD patterns of MTV-MIL-100(Ti,Co) before and after catalysis.

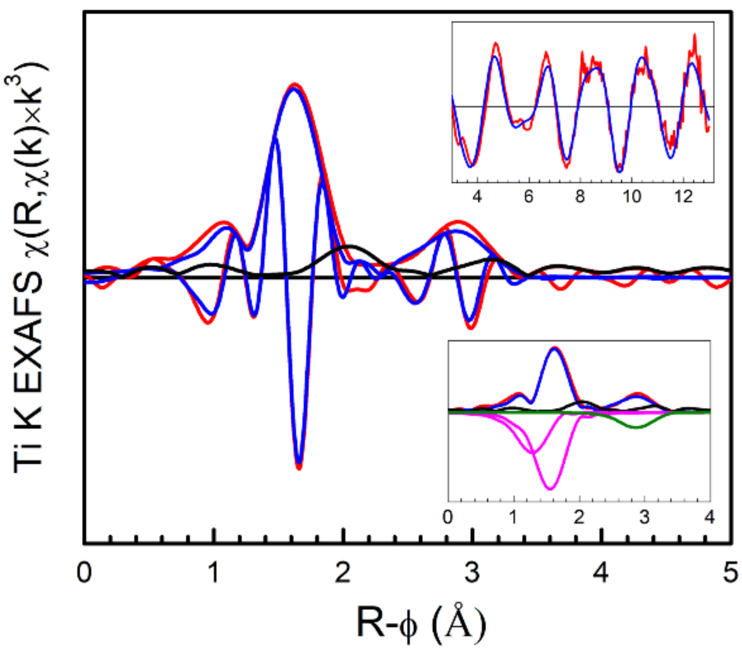

Figure S31. The Fourier transformed spectrum experimental data and fit along with k3-weighted EXAFS data of the MOF. Red line: data, Blue line: fitting.

Table S1. Crystallographic data and Rietveld refinement result of MTV-MIL-100(Ti,Co).

Chemical formula	$C_{4896}Co_{538.56}O_{4352}Ti_{277.44}$
Formula weight	173451.39
Crystal system	Cubic
Space group	*Fd*-3*m* (No. 227)
a/Å	73.6449(1)
Z	1
Temperature/K	298(2)
Wavelength/Å	0.412836
2*θ* range/°	3.00047 – 45.00424
R_{wp}	0.1878
GOOF	3.562

Table S2. Relative spin states energies.

Spin state	Energy (kcal mol^{-1})
Octet	0.0
Sextet	5.1
Quartet	4.8
Doublet	13.7
Doublet BS	0.9

Table S3. Comparisons of photocatalytic activity of MTV-MIL-100(Ti,Co) with previously reported photocatalysts in hydrolysis of AB.

Catalysis	AB conc. (mg mL^{-1})	Temp (K)	Light source	TOF	Refs
MTV-MIL-100 (Ti, Co)	2.86	298	Visible Light	113.7 mol_{H2} $g_{cat.}^{-1}$ min^{-1}	This work
$Cu_{0.55}Ni_{0.45}/TiO_2CdS$ NTs	2.0	298	Visible Light	25.9 mol_{H2} $mol_{cat.}^{-1}$ min^{-1}	27
Ni_2P	17.6	298	Visible Light	82.7 min^{-1}	28
NiCu/CNS	4.0	298	Visible Light	30.6 min^{-1}	29
Co/Cu-190	0.53	298	420nm	68.2 min^{-1}	30
CuCo/g-C_3N_4-1	15.08	298	Visible Light	75.1 min^{-1}	31
Co/C_3N_4-580	21.11	298	Visible Light	93.8 min^{-1}	32
Ni/g-C_3N_4	3.43	298	Visible Light	18.7 mol_{H2} $mol_{cat.}^{-1}$ min^{-1}	33
Co/CoFe-Mo LDH	21.11	298	Visible Light	113.2 min^{-1}	34

NT = nanotube; CNS = carbon nitride nanosheets; LDH = layered double hydroxide

Table S4. The refined and optimized structural parameters of MTV-MIL-100(Ti,Co) from the EXAFS analysis.

Scattering paths	N	R (Å)	σ^2 ($Å^2$)
Ti-O (I)	3.6 ± 0.8	1.9 ± 0.15	0.053 ± 0.02
Ti-O (II)	1.9 ± 0.5	1.7 ± 0.02	0.075 ± 0.01
Ti-M (M = Co/Ti)	1.2 ± 0.3	3.3 ± 0.02	0.073 ± 0.01

Table S5. The *xyz* coordinates used for computational analysis.

Atom	*x*	*y*	*z*
Spin states model- octet			
36			
scf done: -5053.203758			
C	-65.3399	-17.5179	23.04192
C	-67.0259	-15.6779	17.73459
C	-67.0039	-13.6132	20.90976
C	-65.4407	-19.3203	19.75175
C	-62.53	-14.5623	21.35808
C	-62.574	-16.1385	18.00536
Ti	-64.7309	-14.5781	19.3166
Co	-66.9829	-16.8162	20.60551

Co	-63.5336	-17.3121	20.73591
O	-67.5212	-14.7244	21.00353
O	-65.1159	-16.0198	20.15325
O	-66.4361	-18.6839	20.14949
O	-68.7119	-17.1262	20.996
O	-67.362	-16.5467	18.53858
O	-66.3196	-16.9272	22.5435
O	-65.9321	-13.304	20.27872
O	-64.2399	-18.9474	19.74158
O	-66.158	-14.7525	17.89802
O	-64.3025	-17.9337	22.46598
O	-63.4575	-15.2365	17.87843
O	-62.5373	-15.7535	21.67701
O	-62.3668	-16.8459	19.00202
O	-63.1533	-14.0248	20.38159
O	-64.3893	-12.6699	18.11533
O	-61.7932	-18.6343	20.96589
H	-65.3874	-17.6994	24.14049
H	-61.9183	-13.8601	21.96332
H	-67.5212	-15.673	16.73824
H	-67.5012	-12.7522	21.4085
H	-61.9251	-16.2877	17.11501
H	-65.6413	-20.3452	19.36519
H	-69.1836	-16.3474	21.32273
H	-61.3774	-18.395	20.12385
H	-62.2356	-19.4712	20.76476
H	-65.1493	-12.0937	18.26975
H	-64.4502	-12.9304	17.18631

Spin states model- sextet

36

scf done: -5053.195674

C	-65.1804	-17.4104	22.96336
C	-67.106	-15.5395	17.61826
C	-67.0184	-13.8436	20.91151
C	-65.4218	-19.2867	19.65833
C	-62.5364	-14.4652	21.18167
C	-62.5459	-16.2068	17.85269
Ti	-64.7193	-14.6321	19.12304
Co	-66.8388	-16.7889	20.4814
Co	-63.4336	-17.2768	20.56212
O	-67.5408	-14.9691	20.96932
O	-65.1102	-16.1092	19.91518
O	-66.4516	-18.6564	19.98237
O	-68.4344	-17.3869	20.90548

O	-67.4342	-16.4204	18.40213
O	-66.1569	-16.8285	22.45836
O	-65.955	-13.4993	20.31872
O	-64.2155	-18.947	19.71097
O	-66.1844	-14.6509	17.78019
O	-64.142	-17.8303	22.38481
O	-63.411	-15.2721	17.77873
O	-62.49	-15.659	21.48442
O	-62.3199	-16.931	18.82613
O	-63.2188	-13.9339	20.24001
O	-64.5002	-12.5836	18.20173
O	-61.8612	-18.6723	21.20378
H	-65.2212	-17.5811	24.06509
H	-61.9256	-13.7488	21.77144
H	-67.6397	-15.4589	16.64563
H	-67.559	-13.0257	21.43499
H	-61.9434	-16.3552	16.93157
H	-65.6215	-20.3135	19.27332
H	-68.9152	-16.6156	21.2496
H	-62.122	-19.4253	20.65462
H	-62.283	-18.8554	22.05778
H	-64.4712	-11.9415	18.92389
H	-65.3205	-12.4012	17.72393

Spin states model- quartet

36

scf done: -5053.196037

C	-65.5136	-17.4448	22.94415
C	-67.0433	-15.6718	17.80261
C	-66.9585	-13.7131	20.9077
C	-65.5313	-19.142	19.73097
C	-62.474	-14.521	21.34285
C	-62.4738	-16.1536	18.00807
Ti	-64.6554	-14.5912	19.26768
Co	-66.903	-16.6681	20.55149
Co	-63.4928	-17.2405	20.72885
O	-67.4353	-14.841	21.07472
O	-65.0813	-16.0072	20.11967
O	-66.5438	-18.5049	20.09565
O	-68.6257	-17.1638	20.87944
O	-67.456	-16.4299	18.6882
O	-66.538	-16.9445	22.41094
O	-65.9356	-13.3707	20.22681
O	-64.3199	-18.8144	19.7276
O	-66.1	-14.8188	17.86101

O	-64.4314	-17.7855	22.42799
O	-63.3616	-15.256	17.86411
O	-62.4665	-15.7092	21.67531
O	-62.2723	-16.8463	19.01561
O	-63.1109	-14.0035	20.3639
O	-64.406	-12.6817	18.07149
O	-61.8837	-18.701	21.00886
H	-65.616	-17.5972	24.04172
H	-61.8671	-13.8046	21.93569
H	-67.5661	-15.7345	16.82494
H	-67.4901	-12.8759	21.40677
H	-61.8183	-16.31	17.12421
H	-65.7625	-20.1678	19.36343
H	-68.7249	-17.0328	21.83267
H	-61.3576	-18.4533	20.23433
H	-62.3813	-19.4723	20.69966
H	-65.1753	-12.1243	18.2491
H	-64.4761	-12.9253	17.13838

Spin states model- doublet

36

scf done: -5053.181950

C	-65.3328	-17.5283	22.80992
C	-67.0998	-15.6126	17.80541
C	-66.9597	-13.741	20.95127
C	-65.415	-19.0648	19.59916
C	-62.5252	-14.6049	21.29668
C	-62.5173	-16.0883	17.92411
Ti	-64.6795	-14.5412	19.22008
Co	-66.8455	-16.663	20.52731
Co	-63.4568	-17.1994	20.5164
O	-67.404	-14.8842	21.12703
O	-65.0656	-15.9943	20.03199
O	-66.4412	-18.4732	19.98733
O	-68.5333	-17.2344	20.92037
O	-67.4841	-16.3806	18.69707
O	-66.3702	-16.9853	22.35861
O	-65.9764	-13.3684	20.23648
O	-64.2028	-18.7219	19.62023
O	-66.1542	-14.7623	17.83726
O	-64.265	-17.8256	22.23182
O	-63.363	-15.1383	17.8305
O	-62.5313	-15.8261	21.5198
O	-62.3551	-16.8564	18.8793
O	-63.1522	-13.9845	20.38279

O	-64.4673	-12.6005	18.07984
O	-61.897	-18.6553	21.1564
H	-65.3886	-17.7771	23.89293
H	-61.9008	-13.9789	21.96549
H	-67.6591	-15.6654	16.84726
H	-67.4946	-12.9263	21.48299
H	-61.8557	-16.2194	17.04268
H	-65.6005	-20.0719	19.16493
H	-68.6577	-16.9762	21.84409
H	-62.1455	-19.3739	20.55913
H	-62.3536	-18.876	21.98153
H	-65.288	-12.1	18.17773
H	-64.4107	-12.8227	17.14063

Spin states model- BS doublet

36

scf done: -5053.202343

C	-65.3714	-17.4984	23.03559
C	-67.0045	-15.703	17.73424
C	-67.003	-13.624	20.89669
C	-65.4469	-19.326	19.80805
C	-62.5282	-14.548	21.37476
C	-62.5271	-16.1103	18.0113
Ti	-64.7119	-14.593	19.31723
Co	-66.9956	-16.8076	20.61226
Co	-63.4945	-17.2985	20.73806
O	-67.5189	-14.7369	20.99851
O	-65.0931	-16.042	20.15003
O	-66.43	-18.6682	20.21163
O	-68.73	-17.1268	20.97551
O	-67.3471	-16.5658	18.54298
O	-66.3475	-16.884	22.54507
O	-65.9287	-13.3182	20.274
O	-64.2508	-18.9592	19.74057
O	-66.1458	-14.771	17.90058
O	-64.3514	-17.9254	22.45156
O	-63.4336	-15.2274	17.88663
O	-62.535	-15.7382	21.69519
O	-62.3148	-16.8208	19.00263
O	-63.1633	-14.0149	20.40197
O	-64.3907	-12.6753	18.1219
O	-61.8375	-18.6858	20.87939
H	-65.4246	-17.6832	24.13354
H	-61.9116	-13.8432	21.97129
H	-67.4862	-15.7134	16.73133

H	-67.5106	-12.7623	21.38329
H	-61.8691	-16.2332	17.12378
H	-65.6725	-20.3644	19.47386
H	-69.1724	-16.3733	21.39177
H	-61.3608	-18.3832	20.09142
H	-62.3274	-19.4598	20.56041
H	-65.1543	-12.1069	18.28758
H	-64.4583	-12.9307	17.19198

Model for electronic structure and TDDFT – octet
68
scf done: -6268.596618

C	-65.3803	-17.1557	22.98475
C	-66.9199	-15.5226	17.54299
C	-67.0191	-13.558	20.78571
C	-65.1693	-19.1966	19.79766
C	-62.4933	-14.1675	21.19105
C	-62.4945	-16.0745	17.98284
Ti	-64.6974	-14.4291	19.1721
Co	-66.9025	-16.7334	20.38107
Co	-63.42	-17.0179	20.78458
O	-67.5508	-14.6748	20.71616
O	-65.0383	-15.8676	20.03136
O	-66.2297	-18.5706	19.98947
O	-68.6293	-17.1556	20.65426
O	-67.1912	-16.4596	18.28955
O	-66.3824	-16.7502	22.36144
O	-65.9091	-13.2233	20.23633
O	-63.9908	-18.7785	19.92946
O	-66.1157	-14.5487	17.77409
O	-64.2502	-17.4695	22.53367
O	-63.3896	-15.1784	17.84278
O	-62.4756	-15.339	21.57565
O	-62.2428	-16.6836	19.04056
O	-63.161	-13.685	20.21451
O	-64.3663	-12.4802	18.03959
O	-61.6671	-18.2479	21.25721
H	-65.501	-17.2515	24.08833
H	-61.8642	-13.429	21.73251
H	-67.4085	-15.4842	16.54524
H	-65.2822	-20.2545	19.46998
H	-69.1968	-16.4276	20.94185
H	-61.0394	-17.9736	20.57502
H	-62.0251	-19.0803	20.91339
H	-64.244	-11.7438	18.65277

H	-65.1574	-12.2698	17.52639
C	-61.6907	-16.3946	16.76284
C	-61.8433	-15.6453	15.59385
C	-60.7803	-17.4526	16.79034
C	-61.0675	-15.9258	14.46546
H	-62.5619	-14.8243	15.55718
C	-60.0418	-17.7878	15.65324
H	-60.6486	-18.0408	17.69989
C	-60.1804	-17.008	14.49912
H	-59.5871	-17.2483	13.60891
C	-67.7121	-12.4753	21.5545
C	-67.1538	-11.1991	21.64525
C	-68.9277	-12.7407	22.18931
C	-67.786	-10.1933	22.3807
H	-66.2068	-10.9766	21.15162
C	-69.6035	-11.7331	22.88264
H	-69.3645	-13.7398	22.14113
C	-69.0184	-10.4654	22.9852
H	-69.532	-9.6759	23.54649
C	-61.2674	-15.0718	13.25238
C	-59.1001	-18.9466	15.75447
C	-67.1212	-8.85409	22.43503
C	-70.9105	-12.084	23.52361
O	-62.2679	-14.4366	13.06051
O	-60.2563	-15.0503	12.35811
O	-58.7193	-19.5187	14.59349
O	-58.6874	-19.358	16.80459
O	-67.4792	-8.04165	23.45039
O	-66.3124	-8.497	21.62138
O	-71.7373	-11.0549	23.8073
O	-71.2244	-13.2123	23.78553
H	-68.0086	-8.52146	24.10064
H	-71.4228	-10.2408	23.39373
H	-59.253	-19.1883	13.85925
H	-59.4686	-15.4728	12.72426

Only linker – singlet

21

scf done: -797.289678

C	-67.0412	-13.6049	20.96892
O	-67.5525	-14.6921	21.0318
O	-65.8817	-13.4293	20.31948
C	-67.6636	-12.3978	21.60738
C	-67.1549	-11.1049	21.51044
C	-68.8407	-12.6109	22.33768

C	-67.7971	-10.0269	22.13447
H	-66.2478	-10.8561	20.95007
C	-69.4966	-11.5555	22.96666
H	-69.1938	-13.6467	22.37433
C	-68.9626	-10.2642	22.85903
H	-69.5209	-9.47648	23.37546
C	-67.1619	-8.67761	21.9649
C	-70.7566	-11.6962	23.76984
O	-67.759	-7.63562	22.5603
O	-66.1557	-8.52531	21.32296
O	-71.2734	-10.7543	24.3114
O	-71.294	-12.9206	23.8646
H	-68.5528	-7.90963	23.03811
H	-65.5838	-12.5117	20.37199
H	-70.776	-13.5723	23.37406

References

(1) Welter, E.; Chernikov, R.; Herrmann, M.; Nemausat, R. A Beamline for Bulk Sample X-Ray Absorption Spectroscopy at the High Brilliance Storage Ring PETRA III. *AIP Conf. Proc.* **2019**, *2054* (1), 040002.

(2) Rehr, J.C. and Albers, R. C. Scattering-Matrix Formulation of Curved-Wave Multiple-Scattering Theory : Application to x-Ray- Absorption Fine Structur. **1990**, *8139*, 8139.

(3) Rehr, J. J.; Kas, J. J.; Prange, M. P.; Sorini, A. P.; Takimoto, Y.; Vila, F. Ab Initio Theory and Calculations of X-Ray Spectra. *Comptes Rendus Phys.* **2009**, *10* (6), 548–559.

(4) Munoz, M.; Argoul, P.; Farges, F. Continuous Cauchy Wavelet Transform Analyses of EXAFS Spectra: A Qualitative Approach. *Am. Mineral.* **2003**, *88* (4), 694–700.

(5) Muñoz, M.; Farges, F.; Argoul, P. Continuous Cauchy Wavelet Transform of XAFS Spectra. *Phys. Scr. T* **2005**, *T115*, 221–222.

(6) Babonneau, F.; Doeuff, S.; Leaustic, A.; Sanchez, C.; Cartier, C.; Verdaguer, M. XANES and EXAFS Study of Titanium Alkoxides. *Inorg. Chem.* **1988**, *27* (18), 3166–3172.

(7) Jiang, N.; Su, D.; Spence, J. C. H. Determination of Ti Coordination from Pre-Edge Peaks in Ti K -Edge XANES. *Phys. Rev. B - Condens. Matter Mater. Phys.* **2007**, *76* (21), 1–9.

(8) Phoohinkong, W.; Pavasupree, S.; Wannagon, A.; Sanguanpak, S.; Boonyarattanakalin, K.; Mekprasart, W.; Pecharapa, W. Characterization and X-Ray Absorption Spectroscopy of Ilmenite Nanoparticles Derived from Natural Ilmenite Ore via Acidassisted Mechanical Ball-Milling Process. *Adv. Nat. Sci. Nanosci. Nanotechnol.* **2017**, *8* (3), 035012.

(9) La Fontaine, C.; Barthe, L.; Rochet, A.; Briois, V. X-Ray Absorption Spectroscopy and Heterogeneous Catalysis: Performances at the SOLEIL's SAMBA Beamline. *Catal. Today* **2013**, *205*, 148–158.

(10) Ghaffari, M.; Liu, T.; Huang, H.; Tan, O. K.; Shannon, M. Investigation of Local Structure Effect and X-Ray Absorption Characteristics (EXAFS) of Fe (Ti) K-Edge on Photocatalyst Properties of SrTi (1-x)Fe XO (3-δ). *Mater. Chem. Phys.* **2012**, *136* (2–3), 347–357.

(11) Zhao, Y.; Truhlar, D. G. A New Local Density Functional for Main-Group Thermochemistry, Transition Metal Bonding, Thermochemical Kinetics, and Noncovalent Interactions. *J. Chem. Phys.* **2006**, *125* (19), 194101.

(12) Frisch, M. J.; Trucks, G. W.; Schlegel, H. B.; Scuseria, G. E.; Robb, M. A.; Cheeseman, J. R.; Scalmani, G.; Barone, V.; Mennucci, B.; Petersson, G. A.; Nakatsuji, H.; Caricato, M.; Li, X.; Hratchian, H. P.; Izmaylov, A. F.; Bloino, J.; Zheng, G.; Sonnenberg, J. L.; Hada, M.; Ehara, M.; Toyota, K.; Fukuda, R.; Hasegawa, J.; Ishida, M.; Nakajima, T.; Honda, Y.; Kitao, O.; Nakai, H.; Vreven, T.; Montgomery, J. J. A.; Peralta, J. E.; Ogliaro, F.; Bearpark, M.; Heyd, J. J.; Brothers, E.; Kudin, K. N.; Staroverov, V. N.; Kobayashi, R.; Normand, J.; Raghavachari, K.; Rendell, A.; Burant, J. C.; Iyengar, S. S.; Tomasi, J.; Cossi, M.; Rega, N.; Millam, J. M.; Klene, M.; Knox, J. E.; Cross, J. B.; Bakken, V.; Adamo, C.; Jaramillo, J.; Gomperts, R.; Stratmann, R. E.; Yazyev, O.; Austin, A. J.; Cammi, R.; Pomelli, C.; Ochterski, J. W.; Martin, R. L.; Morokuma, K.; Zakrzewski, V. G.; Voth, G. A.; Salvador,

P.; Dannenberg, J. J.; Dapprich, S.; Daniels, A. D.; Farkas, Ö.; Foresman, J. B.; Ortiz, J. V.; Cioslowski, J.; Fox, D. J. Gaussian 09, Revision E.01; Gaussian, Inc.: Wallingford, CT, **2009**.
(13) Weigend, F. Accurate Coulomb-Fitting Basis Sets for H to Rn. *Phys. Chem. Chem. Phys.* **2006**, *8* (9), 1057–1065.
(14) Weigend, F.; Ahlrichs, R. Balanced Basis Sets of Split Valence, Triple Zeta Valence and Quadruple Zeta Valence Quality for H to Rn: Design and Assessment of Accuracy. *Phys. Chem. Chem. Phys.* **2005**, *7* (18), 3297–3305.
(15) Becke, A. D. Density-functional Thermochemistry. III. The Role of Exact Exchange. *J. Chem. Phys.* **1993**, *98* (7), 5648–5652.
(16) Krukau, A. V.; Vydrov, O. A.; Izmaylov, A. F.; Scuseria, G. E. Influence of the Exchange Screening Parameter on the Performance of Screened Hybrid Functionals. *J. Chem. Phys.* **2006**, *125* (22), 224106.
(17) Mennucci, B.; Cancès, E.; Tomasi, J. Evaluation of Solvent Effects in Isotropic and Anisotropic Dielectrics and in Ionic Solutions with a Unified Integral Equation Method: Theoretical Bases, Computational Implementation, and Numerical Applications. *J. Phys. Chem. B* **1997**, *101* (49), 10506–10517.
(18) Scalmani, G.; Frisch, M. J. Continuous Surface Charge Polarizable Continuum Models of Solvation. I. General Formalism. *J. Chem. Phys.* **2010**, *132* (11), 114110.
(19) Wu, X.-P.; Gagliardi, L.; Truhlar, D. G. Cerium Metal–Organic Framework for Photocatalysis. *J. Am. Chem. Soc.* **2018**, *140* (25), 7904–7912.
(20) Aijaz, A.; Karkamkar, A.; Choi, Y. J.; Tsumori, N.; Rönnebro, E.; Autrey, T.; Shioyama, H.; Xu, Q. Immobilizing Highly Catalytically Active Pt Nanoparticles inside the Pores of Metal–Organic Framework: A Double Solvents Approach. *J. Am. Chem. Soc.* **2012**, *134* (34), 13926–13929.
(21) Fu, F.; Wang, C.; Wang, Q.; Martinez-Villacorta, A. M.; Escobar, A.; Chong, H.; Wang, X.; Moya, S.; Salmon, L.; Fouquet, E.; Ruiz, J.; Astruc, D. Highly Selective and Sharp Volcano-Type Synergistic Ni2Pt@ZIF-8-Catalyzed Hydrogen Evolution from Ammonia Borane Hydrolysis. *J. Am. Chem. Soc.* **2018**, *140* (31), 10034–10042.
(22) Lv, H.; Wei, R.; Guo, X.; Sun, L.; Liu, B. Synergistic Catalysis of Binary RuP Nanoclusters on Nitrogen-Functionalized Hollow Mesoporous Carbon in Hydrogen Production from the Hydrolysis of Ammonia Borane. *J. Phys. Chem. Lett.* **2021**, *12* (1), 696–703.
(23) Wang, L.; Li, H.; Zhang, W.; Zhao, X.; Qiu, J.; Li, A.; Zheng, X.; Hu, Z.; Si, R.; Zeng, J. Supported Rhodium Catalysts for Ammonia–Borane Hydrolysis: Dependence of the Catalytic Activity on the Highest Occupied State of the Single Rhodium Atoms. *Angew. Chem. Int. Ed.* **2017**, *56* (17), 4712–4718.
(24) Zhang, M.; Chen, Y.; Chang, J.-N.; Jiang, C.; Ji, W.-X.; Li, L.-Y.; Lu, M.; Dong, L.-Z.; Li, S.-L.; Cai, Y.-P.; Lan, Y.-Q. Efficient Charge Migration in Chemically-Bonded Prussian Blue Analogue/CdS with Beaded Structure for Photocatalytic H2 Evolution. *JACS Au* **2021**, *1* (2), 212–220.
(25) Raza, N.; Raza, W.; Gul, H.; Azam, M.; Lee, J.; Vikrant, K.; Kim, K.-H. Solar-Light-Active Silver Phosphate/Titanium Dioxide/Silica Heterostructures for Photocatalytic Removal of Organic Dye. *J. Clean. Prod.* **2020**, *254*, 120031.
(26) Vikrant, K.; Park, C. M.; Kim, K.-H.; Kumar, S.; Jeon, E.-C. Recent Advancements in Photocatalyst-Based Platforms for the Destruction of Gaseous Benzene: Performance Evaluation of Different Modes of Photocatalytic Operations and against Adsorption Techniques. *J. Photochem. Photobiol. C Photochem. Rev.* **2019**, *41*, 100316.
(27) Sun, D.; Hao, Y.; Wang, C.; Zhang, X.; Yu, X.; Yang, X.; Li, L.; Lu, Z.; Shang, W. TiO2–CdS Supported CuNi Nanoparticles as a Highly Efficient Catalyst for Hydrolysis of Ammonia Borane under Visible-Light Irradiation. *Int. J. Hydrog. Energy* **2020**, *45* (7), 4390–4402.
(28) Song, J.; Gu, X.; Zhang, H. Electrons and Hydroxyl Radicals Synergistically Boost the Catalytic Hydrogen Evolution from Ammonia Borane over Single Nickel Phosphides under Visible Light Irradiation. *ChemistryOpen* **2020**, *9* (3), 366–373.
(29) Zhang, S.; Li, M.; Li, L.; Dushimimana, F.; Zhao, J.; Wang, S.; Han, J.; Zhu, X.; Liu, X.; Ge, Q.; Wang, H. Visible-Light-Driven Multichannel Regulation of Local Electron Density to Accelerate Activation of O–H and B–H Bonds for Ammonia Borane Hydrolysis. *ACS Catal.* **2020**, *10* (24), 14903–14915.
(30) Xu, Y.; Zhang, H.; Song, J.; Wang, D.; Gu, X. Boosting the On-Demand Hydrogen Generation from Aqueous Ammonia Borane by the Visible-Light-Driven Synergistic Electron Effect in Antenna-Reactor-Type Catalysts with Plasmonic Copper Spheres and Noble-Metal-Free Nanoparticles. *Chem. Eng. J.* **2020**, *401*, 126068.
(31) Zhang, H.; Gu, X.; Liu, P.; Song, J.; Cheng, J.; Su, H. Highly Efficient Visible-Light-Driven Catalytic Hydrogen Evolution from Ammonia Borane Using Non-Precious Metal Nanoparticles Supported by Graphitic Carbon Nitride. *J. Mater. Chem. A* **2017**, *5* (5), 2288–2296.

(32) Zhang, H.; Gu, X.; Song, J.; Fan, N.; Su, H. Non-Noble Metal Nanoparticles Supported by Postmodified Porous Organic Semiconductors: Highly Efficient Catalysts for Visible-Light-Driven On-Demand H2 Evolution from Ammonia Borane. *ACS Appl. Mater. Interfaces* **2017**, *9* (38), 32767–32774.
(33) Gao, M.; Yu, Y.; Yang, W.; Li, J.; Xu, S.; Feng, M.; Li, H. Ni Nanoparticles Supported on Graphitic Carbon Nitride as Visible Light Catalysts for Hydrolytic Dehydrogenation of Ammonia Borane. *Nanoscale* **2019**, *11* (8), 3506–3513.
(34) Zhang, S.; Xu, J.; Cheng, H.; Zang, C.; Bian, F.; Sun, B.; Shen, Y.; Jiang, H. Photocatalytic H2 Evolution from Ammonia Borane: Improvement of Charge Separation and Directional Charge Transmission. *ChemSusChem* **2020**, *13* (19), 5264–5272.
